# The enteric nervous system in gastrointestinal disease etiology

**DOI:** 10.1007/s00018-021-03812-y

**Published:** 2021-03-26

**Authors:** Amy Marie Holland, Ana Carina Bon-Frauches, Daniel Keszthelyi, Veerle Melotte, Werend Boesmans

**Affiliations:** 1 Department of Pathology, GROW-School for Oncology and Developmental Biology, Maastricht University Medical Center, Maastricht, The Netherlands; 2 Biomedical Research Institute (BIOMED), Hasselt University, Diepenbeek, Belgium; 3 Department of Internal Medicine, Division of Gastroenterology-Hepatology, NUTRIM-School of Nutrition and Translational Research in Metabolism, Maastricht University Medical Center, Maastricht, The Netherlands; 4 Department of Clinical Genetics, Erasmus MC University Medical Center, Rotterdam, The Netherlands

**Keywords:** Neural crest, Irritable bowel syndrome, Inflammatory bowel disease, Hirschsprung disease, Microbiota

## Abstract

A highly conserved but convoluted network of neurons and glial cells, the enteric nervous system (ENS), is positioned along the wall of the gut to coordinate digestive processes and gastrointestinal homeostasis. Because ENS components are in charge of the autonomous regulation of gut function, it is inevitable that their dysfunction is central to the pathophysiology and symptom generation of gastrointestinal disease. While for neurodevelopmental disorders such as Hirschsprung, ENS pathogenesis appears to be clear-cut, the role for impaired ENS activity in the etiology of other gastrointestinal disorders is less established and is often deemed secondary to other insults like intestinal inflammation. However, mounting experimental evidence in recent years indicates that gastrointestinal homeostasis hinges on multifaceted connections between the ENS, and other cellular networks such as the intestinal epithelium, the immune system, and the intestinal microbiome. Derangement of these interactions could underlie gastrointestinal disease onset and elicit variable degrees of abnormal gut function, pinpointing, perhaps unexpectedly, the ENS as a diligent participant in idiopathic but also in inflammatory and cancerous diseases of the gut. In this review, we discuss the latest evidence on the role of the ENS in the pathogenesis of enteric neuropathies, disorders of gut–brain interaction, inflammatory bowel diseases, and colorectal cancer.

## Introduction

Evolution has endowed the gastrointestinal tract with its own dedicated nervous system. In mammalians, the enteric nervous system (ENS) consists of millions of neurons and glial cells that are organized into interconnected ganglia embedded within the gut wall. The ENS has the ability to autonomously command gastrointestinal tissue dynamics and gut homeostasis, devoid of input from the brain or spinal cord, earning it the *sobriquet* ‘second brain’ [1]. As the greatest division of the autonomic nervous system and rivalling the spinal cord in terms of complexity, ENS components form integrated circuits which independently or in concert with extrinsic parasympathetic and sympathetic innervation regulate a myriad of gut processes including bowel motility, transmucosal movement of fluids, immune responses, and local blood flow [2]. Positioned in the largest sensory organ of the body, the ENS also works alongside the intestinal epithelia, immune system, enteroendocrine system and intestinal microbiome to allow the absorption of nutrients, water, and electrolytes while at the same time to prevent  access to harmful substances present in the lumen [3, 4].

In vertebrates, the majority of enteric neurons and glia develop from neural crest cells originating from the vagal level of the neural tube which invade the foregut and rostro-caudally migrate culminating in the quasi-uniform colonization of the gastrointestinal tract [5, 6]. The constellation of intrinsic primary afferent neurons, interneurons, and motor neurons, with distinct neurochemical coding and functional roles are surrounded by 1–7 times as many enteric glial cells (EGCs) [7] and generally configured within two major ganglionated and interconnected plexus layers: the myenteric and submucosal plexus. Enteric neuron cell bodies are located in the myenteric and submucosal ganglia while their processes extend throughout the external muscle layers, submucosa and mucosa [2]. In addition, EGCs also reside in extraganglionic spaces and are embedded within the intestinal mucosa and muscle layers [7]. Although evidence for functional specialization is rather limited, much like enteric neurons, EGCs can be classified into distinct subtypes based on their morphology and location within the gut wall and along the gastrointestinal tract [8]. Of late, the established classification schemes for ENS cells have been complemented with data from single-cell sequencing analyses that further unravel the identity of enteric neuron and EGC subpopulations [9–13]. The heterogeneous populations of enteric neurons and glia closely interact to coordinate the stereotypic patterns of gut motility and secretion which are key to gastrointestinal homeostasis. To guide some of these processes, the ENS is assisted by different types of enteroendocrine cells, which are scattered throughout the epithelium and monitor the gut lumen [14, 15]. Among these are enterochromaffin cells, a particular serotonin-producing enteroendocrine cell type thought to be crucial for conveying luminal information to the ENS [16–18]. For a detailed presentation on functional circuits and ENS signaling the reader is directed to recent literature [19, 20]. Given its central role as an integrating hub for controlling gastrointestinal physiology [21], it is unsurprising that alterations in ENS function are concomitant with disruptions of gut homeostasis, resulting in both gastrointestinal and extra-gastrointestinal diseases [22, 23]. The complexity of ENS architecture is paralleled by the overarching adverse consequences that can result from perturbations of genes critical for ENS development to subtler alterations in its connectivity and its cross-talk with neighboring cellular systems involved in gut function [24, 25]. In this review, we present the latest evidence for ENS involvement in disorders that strike the gastrointestinal tract. We discuss both experimentally established and hypothesized roles for enteric neurons and EGCs in the pathogenesis of enteric neuropathies, disorders of gut–brain interaction (DGBIs), inflammatory bowel disease (IBD), and colorectal cancer (CRC).

## Enteric neuropathies

Enteric neuropathies emanate from loss, degeneration, and/or functional impairment of enteric neurons [26–28]. They are caused by congenital defects in ENS development, acquired through the effect of infectious agents or toxins, or secondary to other pathological conditions such as diabetes and neurodegenerative disorders [22, 29]. ENS defects vary from subtle alterations in the biochemistry or connectivity within the neural network to a complete loss of ganglia from entire segments of the bowel. The latter of which can result in life-threatening conditions such as Hirschsprung disease (HSCR), where survival of patients depends on early diagnosis and surgical intervention [30, 31].

HSCR is the best known congenital enteric neuropathy affecting 1 in 5000 individuals, in which the absence of ganglia in distal portions of the gut results in difficult expulsion of meconium, causing life-threatening intestinal occlusion, and over time intractable constipation due to the lack of peristalsis in this intestinal region. Collectively, genetic studies of patients with HSCR [32–34] and in vivo transgenic animal models [35–37] have identified multiple genes involved in the development of the ENS, including the receptor tyrosine kinase (*RET*) [38, 39] and endothelin receptor type B (*EDNRB)* [40] and their family members as major players for the HSCR phenotype, together with mutations in *SOX10* [41, 42], *PHOX2B* [43]*,* semaphorins [44, 45], among other genes [46]. Interestingly, recent studies revealed other molecular HSCR candidates [47] and genetic variants, including pathogenic genes, alleles and loci that can exacerbate the susceptibility of HSCR patients in manifesting the disease phenotype [48, 49]. Highly conserved among species, deficiencies along the signaling pathways of these genes may result in failure of ENS progenitors to migrate, proliferate, differentiate or survive within the distal intestine and cause congenital bowel obstruction [5, 50]. Of note, novel work by Chatterjee et al. [51], identified specialized genetic programs active in ENS cells during critical stages of gastrointestinal organogenesis that also control epithelial, endothelial and muscle cell specification. Therefore, such impaired gene networks not only impel ENS precursor cells to colonize the distal gut, causing aganglionosis, but also influence the development of non-neural intestinal tissues [52]. Remarkably, even after the aganglionic intestine has been surgically removed, patients often experience functional abnormalities which fluctuate from severe constipation to fecal incontinence [53]. Although the pathophysiological mechanisms behind these symptoms are not clear, recent work reported histopathological disturbances in the ganglionic bowel of patients who had previously undergone optimal pull-through surgery [54]. Moreover, fatty acid binding protein 7 (FABP7), a marker of immature enteric glia, was significantly upregulated in the myenteric plexus and resulted in a higher ratio of FABP7 to S100 calcium-binding protein B (S100B) expression as compared to controls, signifying a higher proportion of immature EGCs in the ganglionic bowel of HSCR patients [55]. These findings support the notion that megacolon can be associated with impaired differentiation of ENS precursors, or perhaps, with defective enteric gliogenesis. Cumulatively, HSCR substantiates the role of the ENS in survival, as the formation of neural circuits adroit at executing motility need to be prenatally assembled. In the absence of effective interventions, elucidating the subtype composition of enteric ganglia in the ganglionated intestinal segments could help to gain further insight on how to recover aganglionic functionality in HSCR. Additionally, and as the cause of the variable HSCR phenotypes remains elusive, elucidating the contribution of the intestinal milieu for ENS development might indicate that next to genetic and epigenetic factors, environmental cues are also important to determine or attenuate HSCR pathology [56–58]. Indeed, vitamin A deficiency, exposure to high concentrations of drugs such as ibuprofen, mycophenolate, statins, and artemisinin during the critical window of ENS development have been reported to induce a HSCR phenotype in animal models [50]. Also, alterations in epithelial integrity, in the mucosal immune system and in gut microbiota have been put forward as plausible candidates contributing to HSCR [59]. Although direct evidence for a role of the immune system in the onset of HSCR is absent, *RET* and its pathway members are expressed by type 3 innate lymphoid cells (ILC3) and are involved in the organogenesis of intestinal Payer’s patches [60, 61]. Additionally, while it is clear that HSCR patients present with intestinal dysbiosis [62], further investigation would enlighten the paradoxical proposition of whether dysbiosis can contribute to HSCR, or if it is a consequence of impaired elimination mechanisms due to defective intestinal motility [63, 64]. The role of microbes in HSCR pathogenesis is intriguing, yet not entirely surprising, since collective efforts have explored and divulged the important contribution of the gut microbiome in fine-tuning the ENS development and homeostasis [65–72], and vice versa [73].

While significant progress has been made in the genetics and pathophysiology of ENS constituents in HSCR, a detailed understanding of the role of ENS defects in other gastrointestinal disorders sometimes classified as enteric neuropathies remains limited, and their etiology in many cases is defined as idiopathic. For example, a prominent reduction in enteric neuronal cells in the lower esophagus, especially of inhibitory neurons can be a primary cause of achalasia, which is characterized by an absence of esophageal peristalsis and failure of the lower esophageal sphincter to relax upon swallowing [74, 75]. The neuronal loss is believed to be caused by an autoimmune reaction, possibly to a viral infection, in patients with a particular immunogenetic background, and biopsies from patients with achalasia displayed marked immune cell infiltration within the myenteric plexus and increased production of autoantibodies [76–78]. Moreover, genetic factors such as polymorphisms of the protein tyrosine phosphatase N22 (PTPN22), interleukin-23 receptor (IL-23R), and interleukin-10 (IL-10) promoters related to immune cell regulation were reported to contribute to the heterogeneity of disease pathogenesis [79]. Another enteric neuropathy, gastroparesis, is characterized by delayed gastric emptying in the absence of mechanical gastric outlet obstruction. Gastroparesis can be caused by complications of diabetes, and less commonly by medications and surgical interventions but the overwhelming majority of cases remains idiopathic [80, 81]. Studies using human gastric biopsies found a pronounced reduction in interstitial cells of Cajal (ICCs) and neuronal fibers in the circular muscle layer associated with increased immune cell infiltration in the myenteric plexus [82]. Whether gastroparesis can, therefore, be considered as a macrophage-driven ‘cajalopathy’, needs further experimental confirmation [83]. The initiation and progression of chronic intestinal pseudo-obstruction (CIPO), which is characterized by inefficient intestinal transit without any physical obstruction, may involve multiple congenital, acquired or idiopathic causes and distinct cell types including smooth muscle and nerve cells [84]. CIPO is associated with the absence of normal migrating motor complexes that, depending on the underlying pathomechanism, follow a particular neuropathic or myopathic pattern. It can result from an underlying non-gastrointestinal disorder or condition, including a wide variety of systemic, metabolic and organic diseases, such collagen vascular diseases, neurological disorders, or as paraneoplastic phenomenon caused by tumor-derived auto-antibodies [85]. Although CIPO mostly occurs in its sporadic form, it may be also associated with familial inheritance in other patients. For instance, CIPO symptoms can manifest as a result of recessive mutations in important mitochondrial genes (*TYMP, POLG*) [86, 87]; can be caused by mutations in *SGOL1* in chronic atrial and intestinal dysrhythmia (CAID) [88]; and mutations in the *ACTG2* gene in megacystis-microcolon-intestinal hypoperistalsis syndrome (MMIHS) [89]. Additionally, CIPO has been reported in patients with autosomal dominant mutations in the *SOX10* gene in Waardenburg-Shah syndrome [90], and is also related to sex chromosome inheritance in Xq28 with mutations in *Filamin A* and *L1CAM* genes [91, 92]. Although abnormalities in gastrointestinal motility in patients with CIPO manifest in the presence of enteric ganglia [85], a neuronal deficit of approximately 50% associated with increased distance between ganglia, neuronal swelling and axonal degeneration and which correlates with the degree of symptom severity has been reported [93]. This is also the case for slow transit constipation, defined by reduced and infrequent intestinal transit, sensation of anorectal obstruction and solid fecal content [94]. Until now, defining the pathophysiology of slow transit constipation has been challenging as subtle alterations in the ENS are not necessarily detected. Nevertheless, a significant reduction in the size and number of enteric ganglia, and in the number of EGCs and neurons, atypical influx of lymphocytes to the ganglia, decreased numbers of ICCs, presence of intestinal neuronal dysplasia type B, and reduced expression of neurochemical markers [mainly vasoactive intestinal polypeptide (VIP) and Substance P (SP)] have been described in slow transit constipation [29, 95].

Several other neuropathies such as diabetic neuropathy, Chagas disease, hypertrophic pyloric stenosis, intestinal neuronal dysplasia, toxic megacolon and internal anal sphincter achalasia affect the ENS [26, 29, 96] but because of their clear secondary origin are not discussed in this review. Also, neurodegenerative diseases including Alzheimer’s and Parkinson’s disease, and neurodevelopmental disorders such as autism spectrum disorder present with gastrointestinal symptoms linked to ENS dysfunction. We guide the interested reader to excellent literature discussing the role of the ENS in these disorders [22].

Regardless of the nature of the disorder, it is indisputable that the ENS performs a major role in the progression of enteric neuropathies, displaying mild to profound alterations in its structure and causing modest to severe symptoms. However, for most enteric neuropathies other than HSCR, it still remains elusive whether the ENS itself has the ability to be the sole participant in disease initiation. Moreover, as preeminent performers of ENS physiology, studies on the influence of EGCs on the etiology of enteric neuropathies remain undervalued and scarce. Advanced high throughput technology has recently provided novel perspectives on possible functions the ENS may portray in disturbing gastrointestinal homeostasis [12, 48], for instance by expressing disease risk genes and through interactions with other cellular systems.

## Disorders of gut–brain interaction

Disorders of gut–brain interaction (DGBIs), previously more commonly known as functional gastrointestinal disorders (FGIDs), including functional dyspepsia (FD) and irritable bowel syndrome (IBS), define a spectrum of gastrointestinal disorders associated with chronic or fluctuating gastrointestinal symptoms, such as abdominal pain, diarrhea, constipation, bloating and nausea, without harboring an apparent organic structural or biochemical explanation for these symptoms [97, 98]. With evidence in support of a more organic basis accumulating, the most recent iteration of the Rome diagnostic criteria has facilitated a gradual shift from the ‘functional’ nature of these gastrointestinal disorders. Although the pathogenesis of DGBIs remains ill-defined, dysbiosis, visceral hypersensitivity, intestinal dysmotility, and gut–brain axis dysregulation interplays are likely mechanisms responsible for DGBIs [99–104]. Still, their heterogeneous identity makes it unlikely that disease mechanisms can be narrowed down to a single pathophysiological process. Nevertheless, with 40% of persons worldwide anticipated to meet the criteria for DGBIs [105], DGBIs are the most frequent cause of gastroenterological consultations; thus, resulting in major economic effects on global health care systems [106]. Their symptom complex is indicative of altered ENS function. However, only minor evidence of ENS pathology, such as lymphocyte infiltration in myenteric ganglia and increased neurite density in the intestinal mucosa, as well as production of auto-antibodies against neural antigens have been reported, thwarting efforts to identify their intrinsic neurogenic origin [107–110]. Although a pioneering study by Cirillo and colleagues demonstrated that live cell imaging can be used to probe neuronal function in intestinal biopsies [111], ENS defects are not simple to diagnose if ganglia are present, that is not to say that the presence of ganglia averts gastrointestinal anomalies. Consistent with this idea, the disturbed gastrointestinal function in DGBIs could result from subtle alterations in ENS circuitry that have gone undetected in routine clinical diagnosis [6, 25]. However, to date, only a few studies have focused on mechanistic defects in ENS connectivity that manifest later in ENS ontogeny and are unaccompanied by aganglionosis. Inactivation of *Celsr3,* a planar cell polarity (PCP) gene critical for guidance and directional growth of neural processes during murine embryogenesis, in enteric neural crest cells, leads to moderate disruptions of axonal tract configuration in the mature ENS but elicits profound uncoordinated motor activity analogous of DGBIs [112]. Similar experimental strategies using novel ENS-specific transgenic models hold great promise to further refine our understanding of gut motility deficits that do not typify the downstream aftermath of malfunctioning gangliogenesis. Interesting candidates for such approaches include *NXPH1* (neurexophilin 1), *SLC6A4* (serotonin re-uptake transporter, SERT) *HTR3E* (serotonin receptor type 3E) and *HTR4* (serotonin receptor type 4) [113–119]. Others might be identified from genome-wide association (GWA) studies [120–122]. It is plausible that in this way particular gastrointestinal disorders now labeled as ‘functional’, will eventually be identified as enteric neuropathies.

Whilst the cellular outline of the ENS has mostly been configured by birth, the maturation of the neural circuits is finalized amidst > 100 trillion microbes harbored within the postnatal gut [64]. Although the exact means by which the microbiome shapes ENS circuits are unnamed, it is now well-established that the microbial composition impacts the ENS framework, and that the ENS-microbiome work alongside in pathological conditions. Depletion of microbiota beget reductions in myenteric nerve fiber density, alterations in neurochemical coding, decreased excitability in intrinsic primary afferent neurons, defective EGC networks, changes in neurogenic colonic migrating motor complexes and protracted intestinal transit time [69, 72, 123–126], and these effects can be restored following recolonization of adult germ-free mice with conventional microbiota [65, 69, 72, 126]. The linking of microbiota to ENS regulation of motility is one that appears to be evolutionary conserved and can be traced back to 650 million years ago to cnidarians, such as the Hydra [127, 128]. In germ-free Hydra, there is a reduction in the spontaneous peristaltic movements which are under the regulation of neurons and restoration of these movements are observed following the reconstitution of germ-free Hydra with conventional microbiota [129–131]. These findings illustrate that the ENS has evolved to orchestrate responses from microbes and relay them throughout the gastrointestinal tract to influence gut motility. Altered gastrointestinal motility is an important factor in DGBIs [97] and moreover, gut microbiota dysbiosis has been implicated in the onset and progression of DGBIs [132, 133]. Obata et al. [65], identified that enteric neuron-specific deletion of a microbiota-dependent gene, aryl hydrocarbon receptor (*Ahr*), reduced colonic peristaltic activity akin to microbiota-depleted mice, and supplementation of Ahr ligands rectified intestinal motility; alluding to Ahr as a biosensor in enteric neurons, therein fusing their functional output with the luminal environment. Interestingly, a GWA meta-analysis study identified a role for AHR in the biology of stool frequency, often altered in DGBIs [134], and deficiencies in tryptophan-derived Ahr ligands produced by microbiota may contribute to IBS [135, 136]. Furthermore, together with genetic changes in the serotonergic signaling system (see above), the alterations in enterochromaffin cells observed in IBS patients [137, 138] and the impact of the microbiota on this system [72, 139], highlight the importance of the ENS-microbiome dialogue. A dialogue that if perturbed may contribute to the pathogenesis of DGIBs.

The most well-recognized risk factor for DGBIs is previous acute gastrointestinal infection. Permutations in gut milieu post entero-invasive bacteria evasion are associated with remodeling of the neuronal circuitry [140–142], albeit the underlying mechanisms involved in this process are incompletely understood. Recently, Matheis et al. [143] showed that *Salmonella* enterocolitis induced preferential damage to glutamatergic myenteric neurons expressing specific non-canonical inflammasome machinery and pointed to a neuroprotective role of ENS-associated tissue-resident macrophages mediated through an arginase 1-polyamine axis. As targets of a vagal-cholinergic enteric neuron anti-inflammatory pathway [144], and in keeping with their vital role in ENS survival and function [145, 146], this data touts that muscularis macrophages can temper with the enteric-inflammasome circuit and ergo, prevent remolding of the neurochemical representation of enteric neurons. Defective maturation and altered numbers of macrophages have been documented in IBS [147, 148]. Among other functions, macrophage-colony stimulating factor (M-CSF) governs survival, proliferation, and activation of macrophages, and while interstitial cells of Cajal also produce M-CSF, within the ENS, EGCs generate the vast majority of M-CSF [149–151]. Grubišić et al. [150] hindered enteric glial intercellular communication mitigated through connexin-43 hemichannels in a chronic inflammatory mouse model and found that curbing the EGC-regulated activation of macrophages via M-CSF safeguarded against the development of visceral hypersensitivity, another key mechanism in DGBIs. This provides proof of the importance of EGCs in shaping neuronal transmission through the EGC-macrophage axis. Nevertheless, this does not exclude the odds that EGCs interact with other immune entities to tweak ENS circuitry and their functions likely transcend across other gastrointestinal dysfunctions linked to DGBIs.

Although there is a vast amount of literature focusing on visceral hypersensitivity acting through extrinsic afferent pathways [152–159], inflammatory mediators can also modify ENS components, thereby facilitating the development of DGBIs [160, 161]. Increased infiltration and activation of mast cells [162–165], together with elevated levels of mast cell mediators, including histamine and proteases, but also relevant miRNAs and neurotrophins have been substantiated in DGBIs [166–169]. A positive correlation between the number of mast cells in close contact with enteric neurons and the degree of abdominal pain was demonstrated in IBS patients [170], albeit, this is not necessarily reflective of a causal relationship. Neurons can trigger mast cell degranulation and mediator release by means of neuropeptides such as vasoactive intestinal peptide (VIP) [171] which, in turn, invoke the egress of histamine and other mediators from nearby mast cells [172], thus, driving neurogenic inflammation [173]. Reciprocally, the vast quantities of mast cell-derived mediators in mucosal supernatants obtained from IBS patients duly triggered enteric neuron excitability with a favored heightened activation of submucosal plexus neurons [174–176]. What is more, chronic exposure to mucosal mast cell-derived mediators from IBS patients not only stimulated neuronal activation and sprouting of enteric neurons but also evoked hyperexcitability of visceral and somatic pain pathways [107, 177–180]; this is suggestive of an association between visceral hypersensitivity and long-lasting enteric neuronal plasticity. Visceral afferent sensitization has been shown to act, for example, through changes in the expression and function of transient receptor potential vanilloid receptor type-1 (TRPV1) and t-type calcium Cav3.2 channels [181–183]. However, whether TRPV1, which has been the focus of many studies on visceral hypersensitivity [184], is expressed by enteric neurons is still a matter of debate [185–188]. Recent single-cell mRNA sequencing data and studies using transgenic Trpv1-Cre reporter mice seem to further support the notion that TRPV1 expression observed in the gut wall is of extrinsic neuronal origin [9, 12, 13, 189, 190]. It, therefore, remains to be determined whether submucosal enteric neurons are involved in the reduced abdominal pain and visceral hypersensitivity observed in IBS patients after histamine receptor H1 (H1R) blocking [191]. Of interest, histamine, via H1R, mediated ATP-induced Ca^2+^ responses in EGCs and a reduction in S100B + EGCs in the colonic mucosa of IBS patients was inversely coupled with abdominal pain [192]. This study infers that restoring EGC network activity could provide an effective strategy to combat pain in IBS patients. Additionally, tachykinins, neuropeptides primarily expressed by neurons in the gut [190], are also tied to distortions in both pain transmission as well as intestinal motility [193–196], and inhibition of tachykinin pathways offers a potential target for treatment in IBS patients [197]. A tachykinin receptor named neurokinin-2 receptor (NK2R) is chiefly expressed by enteric neurons; and stimulation of NK2R can function as a potent driver of neurogenic inflammation in the ENS through mechanisms that implicate intercellular enteric neuron-glia-nociceptor communication [190]. Mechanistically, antagonizing NK2R signaling restricted the occurrence of a reactive EGC phenotype and increased neurogenic contractions, raising the possibility of a role for EGCs in DGBIs [190, 192].

Going forward, by combining the recent advances that have been made in our understanding of ENS composition and function with the novel findings from DGBIs patient studies [198–201], it should be possible to further elucidate the ENS culprits in DGBIs and bring about therapies targeting the second brain.

## Inflammatory bowel diseases

IBD, a collective term used to describe prolonged inflammation of the gastrointestinal tract, primarily include Crohn’s disease (CD), which can present throughout the entire gastrointestinal tract (the most common localization being the terminal ileum), and ulcerative colitis (UC), presenting in the mucosal layer of the colon. En masse, affecting 2.5–3 million people in Europe [202], IBD is univocally identified as an immune pathology and is believed to develop through interactions between environmental, microbial, and immune-mediated factors in a genetically predisposed host [203, 204]. Nonetheless, it has become apparent that the immune system alone may not account for all aspects of IBD pathology. Neural fiber hypertrophy and hyperplasia in the mucosa, submucosal and myenteric plexus concomitant with infiltration of inflammatory cells, submucosal structural defects, neural fiber retraction and neuromatous lesions have been observed in the inflamed gut of IBD patients. This brought into question whether ENS aberrations are mere epiphenomena related to the inflammatory reaction or that they are involved in IBD pathophysiology, or even, that they can act as predictors of IBD recurrence [205–209]. Echoed by prevailing inflammation-induced derangement of intrinsic neural circuits [210], including neuronal hyperexcitability [211, 212], increased synaptic facilitation [212, 213], reduced descending inhibitory neuromuscular transmission [214–216] and even neuronal death [217], long-lasting intestinal dysmotility can persist ensuing inflammation resolution [218]. Moreover, in vivo studies using transgenic mouse models for enteric hyper- and hypoinnervation indicate that the severity of intestinal inflammation is affected by the density of the enteric neurons [219].

The fact that distinct immune cell subsets of the gut are equipped to respond to neuron-derived signals by expressing neurotransmitter and neuropeptide receptors and inversely, that enteric neurons can respond to inflammatory signals via, for instance, expression of cytokine receptors, posits the existence of functional ENS-immune interactions in the modulation of intestinal inflammation [220–223]. Deciphering these neuro-immune units will likely generate important clues on the role of the ENS in IBD, but will also be relevant for post-infectious DGBIs and those associated with low-grade inflammation. This is elegantly illustrated in a neoteric study from Jarret et al., in which deletion of enteric neuron-derived interleukin-18 (IL-18), a pleiotropic cytokine in intestinal barrier homeostasis and combatting pathogenic infections [224], resulted in increased susceptibility to invasive *Salmonella* typhimurium [225]. Intriguingly, IL-18 is elevated in patients with IBD and anti-IL-18 therapy has been proven efficient to reverse severe gastrointestinal symptoms [226, 227].

Several neuropeptides and neurotransmitters have been shown to govern immunological functions through discrete subsets of immune cells [228]. SP, a highly conserved neuropeptide that mediates ENS signaling as well as immune cell proliferation and cytokine production, is elevated in IBD and there is evidence of increased numbers of SP-positive neurons in the myenteric plexus of UC patients [229–231]. VIP is also involved in a vast number of gastrointestinal functions mediated by the ENS and increased VIP-positive neurons in the submucosal plexus have been documented in CD patients [232, 233]. Notably, mast cells reside in close juxtaposition with intestinal peptide-producing neurons [234] and respond to neuron-derived SP and VIP secretion resulting in mast cell degranulation and cytokine production [235]. Reciprocally, enteric neurons are activated by mast cell-derived mediators inducing neuronal hyperexcitability [236–238]. Conforming to this, higher numbers of mast cells or mast cell mediators in immediate proximity to enteric neurons has been revealed in colonic biopsies of IBD patients relative to controls [239–242], mirroring observations in patients with IBS. Confirmed by live-cell imaging experiments in human intestinal preparations [173], these findings indicate that there is bidirectional communication between mast cells and enteric neurons that might be significant in perpetuating ongoing inflammation.

VIP has also been implicated in the stimulation of innate lymphoid cells (ILC) [243], a rapid-responding, highly abundant group of immune cells found in gut tissues that play a sentinel role in intestinal barrier integrity [244]. Following microbial infection, ILC3, express high levels of the VIP receptor VIPR2, and are the first ILC responders to initiate immune responses in the gastrointestinal tract [245]. Analogously, VIP-producing enteric neurons enhance the release of tissue protective interleukin-22 (IL-22), thereby, serving broadly protective effects on the intestinal epithelium [246]. Genetic deletion of VIP signaling disturbs ILC3 production of IL-22 and leads to increased susceptibility to inflammation in experimental models of colitis [243], and accordingly, dysregulation of ILC3 content and function was also shown in IBD [247]. Enteric cholinergic neurons were also found to promote type 2 inflammation through the release of another highly conserved neuropeptide, neuromedin U (NMU) [248–250]. Interestingly, one of its receptors, NMUR1, is selectively enriched in type 2 ILC (ILC2) and appears to be targeted by NMU-producing cholinergic neurons. The NMU-NMUR1 pathway has been shown to activate ILC2 to produce type 2 effector and tissue-repair cytokines in a Myd88-dependent manner.

Comparable to the NMU-mediated signaling from enteric neurons to ILC2, EGCs can respond to microbial-associated molecular patterns in a Myd88-dependent manner by releasing the RET-ligand glial cell line-derived neurotrophic factor (GDNF), which stimulates neighboring ILC3s-expressing the RET receptor to release IL-22, thus, mediating epithelial repair [251]. This work suggests that, in concert with enteric neurons, EGCs also shape the gastrointestinal immunological environment in the course of gut inflammation and aid in facilitating the maintenance of gut homeostasis. Although in experimental models of colitis and IBD-derived human samples the exact source of GDNF in the gut is not unequivocal, increased GDNF expression showed anti-apoptotic epithelial effects [252, 253], and is strongly upregulated in inflamed areas of CD and UC [252, 254] lending further support to the beneficial role of EGCs in modulating epithelial barrier function. However, reminiscent of astrocytes of the CNS, EGCs also undergo phenotypic alterations in response to persistent hyperinflammatory insults, inter alia an upregulation of glial fibrillary acidic protein (GFAP) [255] and may contribute to the influx of microbial infection by reducing their release of beneficial factors that enhance gut barrier function, such as 15-hydroxyeicosatetraenoic (15-HETE) [256] and S-nitrosogluthathione (GSNO) [257]; although, it is unknown whether this defective gliotransmitter release is causal or consequential of disease progression. Aside from a reduction in beneficial gliotransmission, the gut microenvironment can also shift EGCs towards a phenotype that releases specific gliomediators, such as nitric oxide (NO) [258]. IBD is associated with a discernible NO-dependent inflammatory response within the intestinal milieu; mucosal inducible nitric oxide synthase (iNOS) and neuronal nitric oxide synthase (nNOS) in patients with inflamed and non-inflamed UC were upregulated and downregulated, respectively [259]. Pursuant to this, there have been reports of EGCs coordinating the NO-dependent inflammatory response through the circulation of their signaling molecule, S100B, contending that S100B release may antedate the onset of inflammation [260, 261]. Deconstructing the mechanisms by which EGCs and S100B modulate the inflammatory scenario in IBD warrants further research; however, one emerging theme is through the activation of toll-like receptors (TLRs) [262–265]. Differential expression of TLR subtypes has been reported in IBD patients [266, 267] and strikingly, absence of TLR4, for which polymorphisms have been documented in IBD [268, 269] and which is expressed by enteric neurons and EGCs [270–272], affects S100B and GFAP expression and enhances inhibitory neurotransmission and neuronal death through the interaction of NO with purinergic signaling in murine models [273, 274].

From a clinical standpoint, studies highlighting a mechanistic role for the ENS in IBD are rather scarce. Recent profiling of human intestinal tissue at single-cell resolution discerned that IBD risk genes along with multiple genes related to cytokine signaling were enriched in the ENS [12]. Interestingly, a transcriptomic study from the same laboratory identified differential gene expression in a glial subset present in mucosal biopsies obtained from UC patients and suggests glial involvement in both the regulation of T cells and M-like cells together with changes in tumor necrosis factor (TNF) signaling [275].

It is clear that specific neuro-immune units act to integrate immune control in the intestine, a partnership which can be traced back to organisms as primitive as *C. elegans* [276]. For example, recent work revealed a role for sensory neurons in the regulation of innate immunity during larval development by promoting immune effector transcription, clearance of an intestinal pathogen and resistance to bacterial infection [277]. Increasing evidence also pinpoints the ENS as a crucial player in the interaction between the intestinal microbiome and host defense mechanisms [278], albeit, for now, not in the specific context of IBD. Nonetheless, dysregulation of such interplay likely contributes to intestinal inflammation and further interrogation of the cellular players and mediators perhaps will shed light on the neuro-immune and neuro-microbiome scaffolds conducive to sustaining tissue homeostasis.

## Colorectal cancer

Therapeutic developments and screening measures for early detection have improved survival rates for CRC patients. However, the fact that CRC remains the third most common malignancy and the second leading cause of cancer mortality [279] has prompted for a deeper understanding of cancer progression; shifting the focus from genetic and epigenetic aberrations to the contribution of the tumor microenvironment [280–283]. The composition of the tumor microenvironment contains an assortment of cellular players such as endothelial cells, pericytes, fibroblasts, myofibroblasts, immune cells and neural cells [284, 285]. Although research on the role of nerves in cancer is a growing topic in the oncology field [286] and increased neural markers in colorectal tumors converge with poorer prognosis [287], enteric neurons and glial cells have been largely neglected. The relevance of neural infiltration for tumor progression was first believed to be a passive process in which nerves act as roots for cancer cell migration. This has mainly been studied in the context of perineural invasion (PNI), which in CRC, is associated with a reduced disease-free survival and can serve as an independent prognostic marker for patient outcome [288, 289]. However, active crosstalk contributing to cancer growth, mostly involving paracrine signaling between neurons and cancer cells has been described for several cancer types including pancreatic and gastric cancer [286]. This involves the expression of neurotrophins and axon guidance molecules by cancer cells to induce their own innervation, and active communication from neurons to cancer cells [290–294].

To date, several lines of evidence hint towards a role of the ENS in colorectal carcinogenesis [295, 296]. For instance, the expression of netrin-1, a tumor suppressor protein synthesized by enteric neurons during gastrointestinal organogenesis [297], is found to be reduced in CRC [298, 299]. Also, although the increased cholinergic innervation observed in later phases of CRC indicating poorer prognosis was attributed to parasympathetic extrinsic nerves [300], the extensive population of intrinsic cholinergic neurons could be involved in exacerbating CRC. Interestingly, reduced risk of CRC is observed in patients with megacolon presenting with intrinsic colonic denervation of the intestine [301], suggestive of a protective role of diminished intestinal innervation. This is also in line with a study where chemical ablation of neurons in rats is correlated with a lower incidence of CRC [302]. Proof of tumor epithelial cells docking and migrating along enteric neuronal fibers was reported by Duchalais et al. [303], highlighting the potential of the ENS in steering the migration of CRC. Accounting for the vastness of ENS networks and its extensive interactions with the extrinsic nervous system, it is tentative to speculate that the extra physical support from the ENS could enable tumor cells to travel large ranges and penetrate neighboring tissues, which is consistent with the prognostic value of perineural invasion in CRC [288, 289]. Recent findings by Valès and colleagues indicate that, next to enteric neurons, EGCs could also play a role in the CRC tumor microenvironment [304]. In response to tumor-derived ligands, EGCs converted towards a pro-tumorigenic phenotype, stimulated the activation of cancer stem cells which present tumor-initiating capacities, and in turn promoted tumorigenesis. Additionally, elevated levels of S100B observed in CRC patients might foster a pro-malignant micro-environment, regulating various pro-inflammatory, angiogenic and anti-apoptotic factors [305]. Remodeling of EGCs in the earlier stages of colon carcinogenesis is not unforeseen, weighing the extensive phenotypic plasticity of EGCs in the face of a dynamic environment [8]. Despite preliminary, the above findings herald the ENS as an active player in CRC. From a therapeutic perspective, understanding the molecular mechanisms that allow functional adaptations of ENS signaling during tumorigenesis represents an exciting challenge.

## Conclusions and future perspectives

**Fig. 1:**
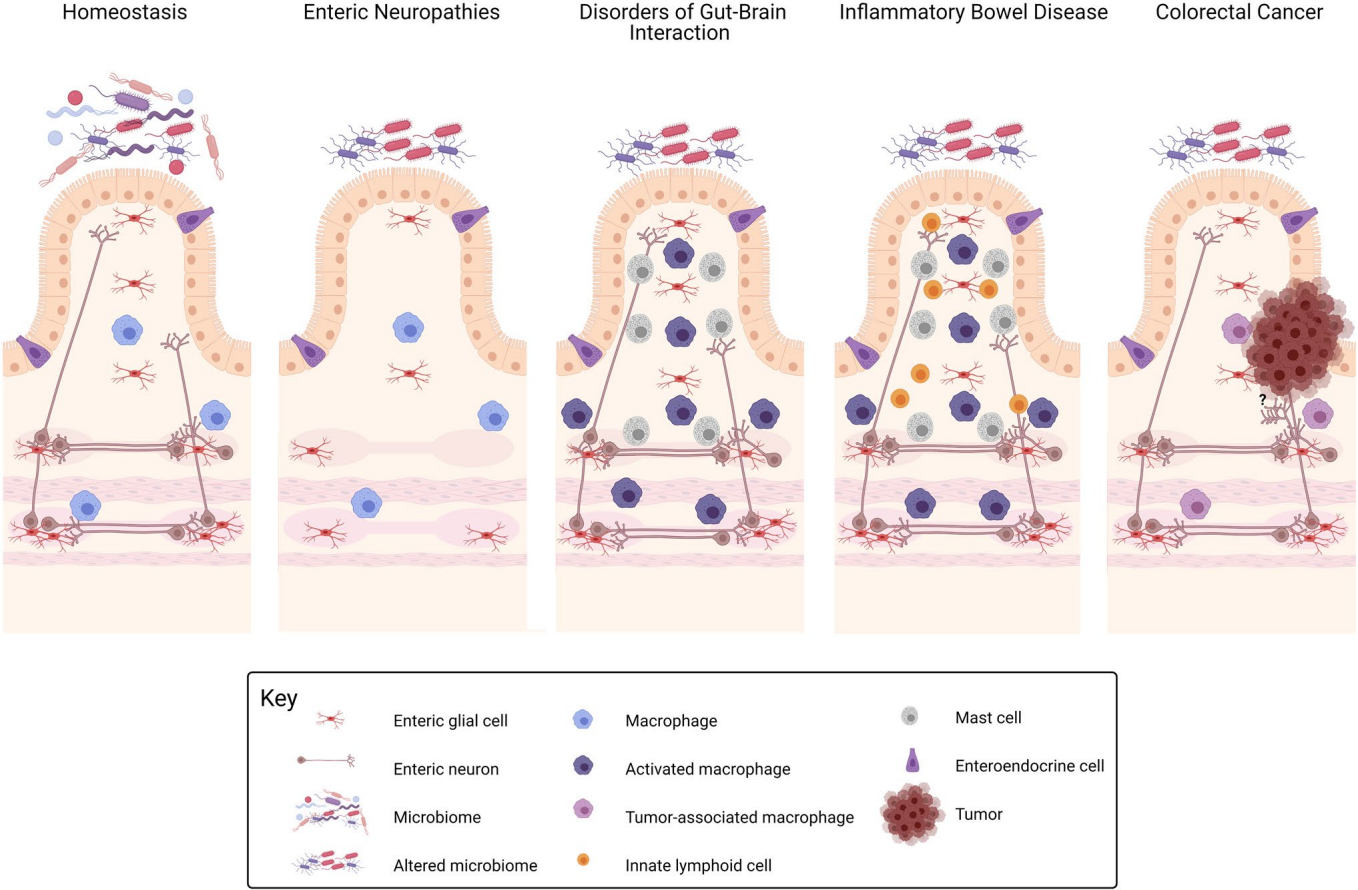
Graphical representation of gastrointestinal disorders from an enteric nervous system perspective. At homeostasis, enteric nervous system (ENS) components (enteric neurons and enteric glial cells) are exposed to and work in concert with the outer and inner microenvironment of the gut to regulate bowel motility, transmucosal movement of fluids, immune responses and local blood flow. Dysfunctions in the ENS may be accountable for a gamut of disorders extending from hypoganglionosis (as observed in some enteric neuropathies) to conditions with a conceivable increased enteric innervation (colorectal cancer). Within this spectrum, subtle changes in ENS circuits as well as alterations in the ENS-immune, ENS-epithelium/enteroendocrine axis and ENS-microbiota axis likely contribute to the pathophysiology of disorders of gut–brain interaction (DGBIs) and inflammatory bowel disorder (IBD). ENS-associated tissue resident macrophages are crucial for normal ENS function and play key roles across the disease spectrum. Defective maturation and altered numbers of macrophages have been documented in DGBIs and IBD. Also, higher mast cell numbers and mediators are present in DGBIs and IBD, while dysregulation of innate lymphoid cells-ENS circuits has been observed in IBD. Disturbances of the physiological microbiome composition can be both a cause or consequence of these disorders. Created with Biorender.com

The precise etiology of enteric neuropathies, IBD, DGBIs and CRC is unknown, and ascertaining if a malfunctioning cell type is the trigger in these disorders or instead a consequence, is not always straightforward. Their heterogeneous presentation and multifactorial nature also withhold us from taking an ENS-centric view. However, alterations in the number of enteric neurons and neuronal subtype composition are implicated in enteric neuropathies, IBD, and DGBIs, though to a lesser extent, alluding that abnormalities in genes or signaling pathways governing ENS circuits are universal across these disorders and may contribute to their pathogenesis. This is corroborated by the concurrence of IBD and IBS, the presence of IBS-like symptoms in IBD patients in apparent remission, the mutual mucosal immune activation and neurochemical coding alterations. Some of which is ultimately reflected in the interchangeable application of in vivo models by researchers investigating both DGBIs and IBD. What is more, while it is well-established that the majority of mutations identified in HSCR are attributed to the RET signaling pathway, antagonizing RET signaling has now also been shown to attenuate post-inflammatory and visceral hypersensitivity in pre-clinical IBS studies [306]. Thus, by impacting motor, sensory and immune functions of the gut, albeit to a variable degree depending on the clinical phenotype, the absence or altered ratio of discrete ENS constituents seems to be a common denominator underpinning the characteristics of these disorders. This leads us to theorize that the ENS may be accountable for a gamut of disorders extending from hypoganglionosis to conditions with an increased enteric neuron density (Fig. 1). Situated within the confines of this spectrum, subtle changes in the connectivity of ENS circuits likely contribute to the pathophysiology of gastrointestinal disorders. However, together with the arcane mechanisms of enteric neuron wiring, the ramifications of faulty ENS connections remain an understudied area. Carving a distinction between the involvement of the ENS in these disorders is quite arduous as the functional-organic dichotomy between some enteric neuropathies, DGBIs and IBD is possibly archaic. Nevertheless, a more comprehensive understanding of ENS development, function and its modes of failure could be crucial in our pursuit of establishing the foundations of some of these diseases and may point towards the endowment of ENS factors responsible for their pathogenesis. Undeniably, gastrointestinal physiology is not solely reliant on a normal ENS but is also influenced by multilateral interactions between the ENS and other systems such as the intestinal epithelia, immune system and microbiome. Evidence gathered in the past few years demonstrates that the neural-immune-microbiome entente, for which the assembly can be retraced to primitive species, is pivotal in a disease context as aberrations in their crosstalk result in variable degrees of abnormal gut function, exemplified in the described disorders.

Unified interdisciplinary efforts will be needed to further disentangle the *Daedalian* biology of the ENS and its cross-talk with other cellular systems [21]. Coupling such insights with in vivo gene editing strategies of candidate genes emanating from recent high-resolution transcriptomic mapping and advances in human intestinal organoids [307, 308] together with novel live-cell imaging modalities [309] will help consolidate the current platforms to model these disorders. In addition, the development of novel ENS-based therapeutic strategies warrants human studies to confirm the clinical relevance of experimental findings.

## References

[1] Gershon MD (1998). The second brain : the scientific basis of gut instinct and a groundbreaking new understanding of nervous disorders of the stomach and intestine.

[2] Furness JB (2012). The enteric nervous system and neurogastroenterology. Nat Rev Gastroenterol Hepatol.

[3] Yoo BB, Mazmanian SK (2017). The enteric network: interactions between the immune and nervous systems of the gut. Immunity.

[4] Margolis KG, Gershon MD, Bogunovic M (2016). Cellular organization of neuroimmune interactions in the gastrointestinal tract. Trends Immunol.

[5] Heanue TA, Pachnis V (2007). Enteric nervous system development and Hirschsprung's disease: advances in genetic and stem cell studies. Nat Rev Neurosci.

[6] Avetisyan M, Schill EM, Heuckeroth RO (2015). Building a second brain in the bowel. J Clin Invest.

[7] Grubisic V, Gulbransen BD (2017). Enteric glia: the most alimentary of all glia. J Physiol.

[8] Boesmans W, Lasrado R, Vanden Berghe P, Pachnis V (2015). Heterogeneity and phenotypic plasticity of glial cells in the mammalian enteric nervous system. Glia.

[9] Zeisel A, Hochgerner H, Lonnerberg P, Johnsson A, Memic F, van der Zwan J, Haring M, Braun E, Borm LE, La Manno G, Codeluppi S, Furlan A, Lee K, Skene N, Harris KD, Hjerling-Leffler J, Arenas E, Ernfors P, Marklund U, Linnarsson S (2018). Molecular architecture of the mouse nervous system. Cell.

[10] May-Zhang AA, Tycksen E, Southard-Smith AN, Deal KK, Benthal JT, Buehler DP, Adam M, Simmons AJ, Monaghan JR, Matlock BK (2020). Combinatorial transcriptional profiling of mouse and human enteric neurons identifies shared and disparate subtypes in situ. Gastroenterology.

[11] Lasrado R, Boesmans W, Kleinjung J, Pin C, Bell D, Bhaw L, McCallum S, Zong H, Luo L, Clevers H, Vanden Berghe P, Pachnis V (2017). Lineage-dependent spatial and functional organization of the mammalian enteric nervous system. Science.

[12] Drokhlyansky E, Smillie CS, Van Wittenberghe N, Ericsson M, Griffin GK, Eraslan G, Dionne D, Cuoco MS, Goder-Reiser MN, Sharova T, Kuksenko O, Aguirre AJ, Boland GM, Graham D, Rozenblatt-Rosen O, Xavier RJ, Regev A (2020). The human and mouse enteric nervous system at single-cell resolution. Cell.

[13] Morarach K, Mikhailova A, Knoflach V, Memic F, Kumar R, Li W, Ernfors P, Marklund U (2021). Diversification of molecularly defined myenteric neuron classes revealed by single-cell RNA sequencing. Nat Neurosci.

[14] Fothergill LJ, Furness JB (2018). Diversity of enteroendocrine cells investigated at cellular and subcellular levels: the need for a new classification scheme. Histochem Cell Biol.

[15] Gribble FM, Reimann F (2016). Enteroendocrine cells: chemosensors in the intestinal epithelium. Annu Rev Physiol.

[16] Mawe GM, Hoffman JM (2013). Serotonin signalling in the gut–functions, dysfunctions and therapeutic targets. Nat Rev Gastroenterol Hepatol.

[17] Alcaino C, Knutson KR, Treichel AJ, Yildiz G, Strege PR, Linden DR, Li JH, Leiter AB, Szurszewski JH, Farrugia G, Beyder A (2018). A population of gut epithelial enterochromaffin cells is mechanosensitive and requires Piezo2 to convert force into serotonin release. Proc Natl Acad Sci.

[18] Bellono NW, Bayrer JR, Leitch DB, Castro J, Zhang C, O'Donnell TA, Brierley SM, Ingraham HA, Julius D (2017). Enterochromaffin cells are gut chemosensors that couple to sensory neural pathways. Cell.

[19] Fung C, Vanden Berghe P (2020). Functional circuits and signal processing in the enteric nervous system. Cell Mol Life Sci.

[20] Spencer NJ, Hu H (2020). Enteric nervous system: sensory transduction, neural circuits and gastrointestinal motility. Nat Rev Gastroenterol Hepatol.

[21] Bon-Frauches AC, Boesmans W (2020). The enteric nervous system: the hub in a star network. Nat Rev Gastroenterol Hepatol.

[22] Rao M, Gershon MD (2016). The bowel and beyond: the enteric nervous system in neurological disorders. Nat Rev Gastroenterol Hepatol.

[23] Wood JD (2016). Enteric nervous system: neuropathic gastrointestinal motility. Dig Dis Sci.

[24] Rao M, Gershon MD (2018). Enteric nervous system development: what could possibly go wrong?. Nat Rev Neurosci.

[25] Gershon MD (2010). Developmental determinants of the independence and complexity of the enteric nervous system. Trends Neurosci.

[26] Pesce M, Borrelli O, Saliakellis E, Thapar N (2018). Gastrointestinal neuropathies: new insights and emerging therapies. Gastroenterol Clin N Am.

[27] Di Nardo G, Blandizzi C, Volta U, Colucci R, Stanghellini V, Barbara G, Del Tacca M, Tonini M, Corinaldesi R, De Giorgio R (2008). Review article: molecular, pathological and therapeutic features of human enteric neuropathies. Aliment Pharmacol Ther.

[28] Brosens E, Burns AJ, Brooks AS, Matera I, Borrego S, Ceccherini I, Tam PK, García-Barceló M-M, Thapar N, Benninga MA, Hofstra RMW, Alves MM (2016). Genetics of enteric neuropathies. Dev Biol.

[29] Knowles CH, Lindberg G, Panza E, De Giorgio R (2013). New perspectives in the diagnosis and management of enteric neuropathies. Nat Rev Gastro Hepat.

[30] Tam PK (2016). Hirschsprung's disease: a bridge for science and surgery. J Pediatr Surg.

[31] McKeown SJ, Stamp L, Hao MM, Young HM (2013). Hirschsprung disease: a developmental disorder of the enteric nervous system. Wiley Interdiscip Rev Dev Biol.

[32] Alves MM, Sribudiani Y, Brouwer RW, Amiel J, Antiñolo G, Borrego S, Ceccherini I, Chakravarti A, Fernández RM, Garcia-Barcelo MM, Griseri P, Lyonnet S, Tam PK, van Ijcken WF, Eggen BJ, te Meerman GJ, Hofstra RM (2013). Contribution of rare and common variants determine complex diseases-Hirschsprung disease as a model. Dev Biol.

[33] Parisi MA, Kapur RP (2000). Genetics of Hirschsprung disease. Curr Opin Pediatr.

[34] Panza E, Knowles CH, Graziano C, Thapar N, Burns AJ, Seri M, Stanghellini V, De Giorgio R (2012). Genetics of human enteric neuropathies. Prog Neurobiol.

[35] Bondurand N, Southard-Smith EM (2016). Mouse models of Hirschsprung disease and other developmental disorders of the enteric nervous system: old and new players. Dev Biol.

[36] Burzynski G, Shepherd IT, Enomoto H (2009). Genetic model system studies of the development of the enteric nervous system, gut motility and Hirschsprung's disease. Neurogastroenterol Motil.

[37] Heanue TA, Pachnis V (2006). Expression profiling the developing mammalian enteric nervous system identifies marker and candidate Hirschsprung disease genes. Proc Natl Acad Sci USA.

[38] Edery P, Pelet A, Mulligan LM, Abel L, Attié T, Dow E, Bonneau D, David A, Flintoff W, Jan D (1994). Long segment and short segment familial Hirschsprung's disease: variable clinical expression at the RET locus. J Med Genet.

[39] Angrist M, Bolk S, Thiel B, Puffenberger EG, Hofstra RM, Buys CH, Cass DT, Chakravarti A (1995). Mutation analysis of the RET receptor tyrosine kinase in Hirschsprung disease. Hum Mol Genet.

[40] Puffenberger EG, Hosoda K, Washington SS, Nakao K, deWit D, Yanagisawa M, Chakravarti A (1994). A missense mutation of the endothelin-B receptor gene in multigenic hirschsprung's disease. Cell.

[41] Sham MH, Lui VC, Fu M, Chen B, Tam PK (2001). SOX10 is abnormally expressed in aganglionic bowel of Hirschsprung's disease infants. Gut.

[42] Herbarth B, Pingault V, Bondurand N, Kuhlbrodt K, Hermans-Borgmeyer I, Puliti A, Lemort N, Goossens M, Wegner M (1998). Mutation of the Sry-related Sox10 gene in Dominant megacolon, a mouse model for human Hirschsprung disease. Proc Natl Acad Sci USA.

[43] Garcia-Barceló M, Sham MH, Lui VCH, Chen BLS, Ott J, Tam PKH (2003). Association study of PHOX2B as a candidate gene for Hirschsprung's disease. Gut.

[44] Luzón-Toro B, Fernández RM, Torroglosa A, de Agustín JC, Méndez-Vidal C, Segura DI, Antiñolo G, Borrego S (2013). Mutational spectrum of semaphorin 3A and semaphorin 3D genes in Spanish Hirschsprung patients. PLoS ONE.

[45] Jiang Q, Arnold S, Heanue T, Kilambi KP, Doan B, Kapoor A, Ling AY, Sosa MX, Guy M, Jiang Q, Burzynski G, West K, Bessling S, Griseri P, Amiel J, Fernandez RM, Verheij JBGM, Hofstra RMW, Borrego S, Lyonnet S, Ceccherini I, Gray JJ, Pachnis V, McCallion AS, Chakravarti A (2015). Functional loss of semaphorin 3C and/or semaphorin 3D and their epistatic interaction with ret are critical to Hirschsprung disease liability. Am J Hum Genet.

[46] Amiel J, Sproat-Emison E, Garcia-Barcelo M, Lantieri F, Burzynski G, Borrego S, Pelet A, Arnold S, Miao X, Griseri P, Brooks AS, Antinolo G, de Pontual L, Clement-Ziza M, Munnich A, Kashuk C, West K, Wong KK, Lyonnet S, Chakravarti A, Tam PK, Ceccherini I, Hofstra RM, Fernandez R (2008). Hirschsprung disease, associated syndromes and genetics: a review. J Med Genet.

[47] Mederer T, Schmitteckert S, Volz J, Martínez C, Röth R, Thumberger T, Eckstein V, Scheuerer J, Thöni C, Lasitschka F, Carstensen L, Günther P, Holland-Cunz S, Hofstra R, Brosens E, Rosenfeld JA, Schaaf CP, Schriemer D, Ceccherini I, Rusmini M, Tilghman J, Luzón-Toro B, Torroglosa A, Borrego S, Sze-Man Tang C, Garcia-Barceló M, Tam P, Paramasivam N, Bewerunge-Hudler M, De La Torre C, Gretz N, Rappold GA, Romero P, Niesler B (2020). A complementary study approach unravels novel players in the pathoetiology of Hirschsprung disease. PLoS Genet.

[48] Tilghman JM, Ling AY, Turner TN, Sosa MX, Krumm N, Chatterjee S, Kapoor A, Coe BP, Nguyen K-DH, Gupta N, Gabriel S, Eichler EE, Berrios C, Chakravarti A (2019). Molecular genetic anatomy and risk profile of Hirschsprung’s disease. N Engl J Med.

[49] Kuil L, MacKenzie KC, Tang CS, Windster JD, Le TL, Karim A, de Graaf BM, van der Helm R, van Bever Y, Sloots CEJ, Meeussen C, Tibboel D, de Klein A, Wijnen RMH, Amiel J, Lyonnet S, Garcia-Barcelo M-M, Tam PKH, Alves MM, Brooks A, Hofstra RMW, Brosens E (2020). Size matters: large copy number losses reveal novel Hirschsprung disease genes. medRxiv.

[50] Heuckeroth RO (2018). Hirschsprung disease—integrating basic science and clinical medicine to improve outcomes. Nat Rev Gastroenterol Hepatol.

[51] Chatterjee S, Nandakumar P, Auer DR, Gabriel SB, Chakravarti A (2019). Gene- and tissue-level interactions in normal gastrointestinal development and Hirschsprung disease. Proc Natl Acad Sci.

[52] Hao MM, Foong JP, Bornstein JC, Li ZL, Vanden Berghe P, Boesmans W (2016). Enteric nervous system assembly: functional integration within the developing gut. Dev Biol.

[53] Dai Y, Deng Y, Lin Y, Ouyang R, Li L (2020). Long-term outcomes and quality of life of patients with Hirschsprung disease: a systematic review and meta-analysis. BMC Gastroenterol.

[54] Kapur RP, Smith C, Ambartsumyan L (2020). Postoperative Pullthrough obstruction in Hirschsprung disease: etiologies and diagnosis. Pediatr Dev Pathol.

[55] Tani G, Tomuschat C, O'Donnell AM, Coyle D, Puri P (2017). Increased population of immature enteric glial cells in the resected proximal ganglionic bowel of Hirschsprung's disease patients. J Surg Res.

[56] Jaroy EG, Acosta-Jimenez L, Hotta R, Goldstein AM, Emblem R, Klungland A, Ougland R (2019). "Too much guts and not enough brains": (epi)genetic mechanisms and future therapies of Hirschsprung disease—a review. Clin Epigenetics.

[57] Obata Y, Pachnis V (2016). The effect of microbiota and the immune system on the development and organization of the enteric nervous system. Gastroenterology.

[58] Torroglosa A, Alves MM, Fernández RM, Antiñolo G, Hofstra RM, Borrego S (2016). Epigenetics in ENS development and Hirschsprung disease. Dev Biol.

[59] Gosain A (2016). Established and emerging concepts in Hirschsprung’s-associated enterocolitis. Pediatr Surg Int.

[60] Ibiza S, García-Cassani B, Ribeiro H, Carvalho T, Almeida L, Marques R, Misic AM, Bartow-McKenney C, Larson DM, Pavan WJ, Eberl G, Grice EA, Veiga-Fernandes H (2016). Glial cell-derived neuroregulators control type 3 innate lymphoid cells and gut defence. Nature.

[61] Veiga-Fernandes H, Coles MC, Foster KE, Patel A, Williams A, Natarajan D, Barlow A, Pachnis V, Kioussis D (2007). Tyrosine kinase receptor RET is a key regulator of Peyer's patch organogenesis. Nature.

[62] Neuvonen MI, Korpela K, Kyrklund K, Salonen A, de Vos W, Rintala RJ, Pakarinen MP (2018). Intestinal microbiota in Hirschsprung disease. J Pediatr Gastroenterol Nutr.

[63] Tang W, Su Y, Yuan C, Zhang Y, Zhou L, Peng L, Wang P, Chen G, Li Y, Li H, Zhi Z, Chang H, Hang B, Mao J-H, Snijders AM, Xia Y (2020). Prospective study reveals a microbiome signature that predicts the occurrence of post-operative enterocolitis in Hirschsprung disease (HSCR) patients. Gut Microbes.

[64] Foong JPP, Hung LY, Poon S, Savidge TC, Bornstein JC (2020). Early life interaction between the microbiota and the enteric nervous system. Am J Physiol-Gastr L.

[65] Obata Y, Castano A, Boeing S, Bon-Frauches AC, Fung C, Fallesen T, de Aguero MG, Yilmaz B, Lopes R, Huseynova A, Horswell S, Maradana MR, Boesmans W, Vanden Berghe P, Murray AJ, Stockinger B, Macpherson AJ, Pachnis V (2020). Neuronal programming by microbiota regulates intestinal physiology. Nature.

[66] Muller PA, Matheis F, Schneeberger M, Kerner Z, Jove V, Mucida D (2020). Microbiota-modulated CART(+) enteric neurons autonomously regulate blood glucose. Science.

[67] Anitha M, Reichardt F, Tabatabavakili S, Nezami BG, Chassaing B, Mwangi S, Vijay-Kumar M, Gewirtz A, Srinivasan S (2016). Intestinal dysbiosis contributes to the delayed gastrointestinal transit in high-fat diet fed mice. Cell Mol Gastroenterol Hepatol.

[68] Cossais F, Durand T, Chevalier J, Boudaud M, Kermarrec L, Aubert P, Neveu I, Naveilhan P, Neunlist M (2016). Postnatal development of the myenteric glial network and its modulation by butyrate. Am J Physiol Gastrointest Liver Physiol.

[69] Kabouridis PS, Lasrado R, McCallum S, Chng SH, Snippert HJ, Clevers H, Pettersson S, Pachnis V (2015). Microbiota controls the homeostasis of glial cells in the gut lamina propria. Neuron.

[70] Collins J, Borojevic R, Verdu EF, Huizinga JD, Ratcliffe EM (2014). Intestinal microbiota influence the early postnatal development of the enteric nervous system. Neurogastroenterol Motil.

[71] Anitha M, Vijay-Kumar M, Sitaraman SV, Gewirtz AT, Srinivasan S (2012). Gut microbial products regulate murine gastrointestinal motility via Toll-like receptor 4 signaling. Gastroenterology.

[72] De Vadder F, Grasset E, Manneras Holm L, Karsenty G, Macpherson AJ, Olofsson LE, Backhed F (2018). Gut microbiota regulates maturation of the adult enteric nervous system via enteric serotonin networks. Proc Natl Acad Sci USA.

[73] Rolig AS, Mittge EK, Ganz J, Troll JV, Melancon E, Wiles TJ, Alligood K, Stephens WZ, Eisen JS, Guillemin K (2017). The enteric nervous system promotes intestinal health by constraining microbiota composition. PLoS Biol.

[74] Rieder E, Fernandez-Becker NQ, Sarosiek J, Guillaume A, Azagury DE, Clarke JO (2020). Achalasia: physiology and diagnosis. Ann N Y Acad Sci.

[75] Boeckxstaens GE (2016). Achalasia: from bench to peroral endoscopic myotomy. Dig Dis.

[76] Moses PL, Ellis LM, Anees MR, Ho W, Rothstein RI, Meddings JB, Sharkey KA, Mawe GM (2003). Antineuronal antibodies in idiopathic achalasia and gastro-oesophageal reflux disease. Gut.

[77] Kraichely RE, Farrugia G, Pittock SJ, Castell DO, Lennon VA (2010). Neural autoantibody profile of primary achalasia. Dig Dis Sci.

[78] Ganem D, Kistler A, DeRisi J (2010). Achalasia and viral infection: new insights from veterinary medicine. Sci Transl Med.

[79] Pressman A, Behar J (2017). Etiology and pathogenesis of idiopathic achalasia. J Clin Gastroenterol.

[80] Camilleri M, Chedid V, Ford AC, Haruma K, Horowitz M, Jones KL, Low PA, Park SY, Parkman HP, Stanghellini V (2018). Gastroparesis. Nat Rev Dis Primers.

[81] Grover M, Farrugia G, Stanghellini V (2019). Gastroparesis: a turning point in understanding and treatment. Gut.

[82] Grover M, Farrugia G, Lurken MS, Bernard CE, Faussone-Pellegrini MS, Smyrk TC, Parkman HP, Abell TL, Snape WJ, Hasler WL, Ünalp-Arida A, Nguyen L, Koch KL, Calles J, Lee L, Tonascia J, Hamilton FA, Pasricha PJ (2011). Cellular changes in diabetic and idiopathic gastroparesis. Gastroenterology.

[83] Pasricha PJ, Grover M, Yates KP, Abell TL, Bernard CE, Koch KL, McCallum RW, Sarosiek I, Kuo B, Bulat R, Chen J, Shulman R, Lee L, Tonascia J, Miriel LA, Hamilton F, Farrugia G, Parkman HP (2021). Functional dyspepsia and gastroparesis in tertiary care are interchangeable syndromes with common clinical and pathological features. Gastroenterology.

[84] De Giorgio R, Cogliandro RF, Barbara G, Corinaldesi R, Stanghellini V (2011). Chronic intestinal pseudo-obstruction: clinical features, diagnosis, and therapy. Gastroenterol Clin North Am.

[85] Downes TJ, Cheruvu MS, Karunaratne TB, De Giorgio R, Farmer AD (2018). Pathophysiology, diagnosis, and management of chronic intestinal pseudo-obstruction. J Clin Gastroenterol.

[86] Van Goethem G, Schwartz M, Löfgren A, Dermaut B, Van Broeckhoven C, Vissing J (2003). Novel POLG mutations in progressive external ophthalmoplegia mimicking mitochondrial neurogastrointestinal encephalomyopathy. Eur J Hum Genet.

[87] Hunter MF, Peters H, Salemi R, Thorburn D, Mackay MT (2011). Alpers syndrome with mutations in POLG: clinical and investigative features. Pediatr Neurol.

[88] Chetaille P, Preuss C, Burkhard S, Côté JM, Houde C, Castilloux J, Piché J, Gosset N, Leclerc S, Wünnemann F, Thibeault M, Gagnon C, Galli A, Tuck E, Hickson GR, El Amine N, Boufaied I, Lemyre E, de Santa BP, Faure S, Jonzon A, Cameron M, Dietz HC, Gallo-McFarlane E, Benson DW, Moreau C, Labuda D, Zhan SH, Shen Y, Jomphe M, Jones SJ, Bakkers J, Andelfinger G (2014). Mutations in SGOL1 cause a novel cohesinopathy affecting heart and gut rhythm. Nat Genet.

[89] Thorson W, Diaz-Horta O, Foster J, Spiliopoulos M, Quintero R, Farooq A, Blanton S, Tekin M (2014). De novo ACTG2 mutations cause congenital distended bladder, microcolon, and intestinal hypoperistalsis. Hum Genet.

[90] Pingault V, Girard M, Bondurand N, Dorkins H, Van Maldergem L, Mowat D, Shimotake T, Verma I, Baumann C, Goossens M (2002). SOX10 mutations in chronic intestinal pseudo-obstruction suggest a complex physiopathological mechanism. Hum Genet.

[91] Gargiulo A, Auricchio R, Barone MV, Cotugno G, Reardon W, Milla PJ, Ballabio A, Ciccodicola A, Auricchio A (2007). Filamin A is mutated in X-linked chronic idiopathic intestinal pseudo-obstruction with central nervous system involvement. Am J Hum Genet.

[92] Deglincerti A, De Giorgio R, Cefle K, Devoto M, Pippucci T, Castegnaro G, Panza E, Barbara G, Cogliandro RF, Mungan Z, Palanduz S, Corinaldesi R, Romeo G, Seri M, Stanghellini V (2007). A novel locus for syndromic chronic idiopathic intestinal pseudo-obstruction maps to chromosome 8q23-q24. Eur J Hum Genet.

[93] Boschetti E, Malagelada C, Accarino A, Malagelada JR, Cogliandro RF, Gori A, Bonora E, Giancola F, Bianco F, Tugnoli V, Clavenzani P, Azpiroz F, Stanghellini V, Sternini C, De Giorgio R (2019). Enteric neuron density correlates with clinical features of severe gut dysmotility. Am J Physiol Gastrointest Liver Physiol.

[94] Bharucha AE, Lacy BE (2020). Mechanisms, Evaluation, and Management of Chronic Constipation. Gastroenterology.

[95] Bassotti G, Villanacci V, Creţoiu D, Creţoiu SM, Becheanu G (2013). Cellular and molecular basis of chronic constipation: taking the functional/idiopathic label out. World J Gastroenterol.

[96] Khoury-Hanold W, Yordy B, Kong P, Kong Y, Ge W, Szigeti-Buck K, Ralevski A, Horvath Tamas L, Iwasaki A (2016). Viral spread to enteric neurons links genital HSV-1 infection to toxic megacolon and lethality. Cell Host Microbe.

[97] Black CJ, Drossman DA, Talley NJ, Ruddy J, Ford AC (2020). Functional gastrointestinal disorders: advances in understanding and management. The Lancet.

[98] Drossman DA (2016). Functional gastrointestinal disorders: history, pathophysiology, clinical features, and Rome IV. Gastroenterology.

[99] Enck P, Aziz Q, Barbara G, Farmer AD, Fukudo S, Mayer EA, Niesler B, Quigley EM, Rajilic-Stojanovic M, Schemann M, Schwille-Kiuntke J, Simren M, Zipfel S, Spiller RC (2016). Irritable bowel syndrome. Nat Rev Dis Primers.

[100] Ford AC, Mahadeva S, Carbone MF, Lacy BE, Talley NJ (2020). Functional dyspepsia. The Lancet.

[101] Ford AC, Sperber AD, Corsetti M, Camilleri M (2020). Irritable bowel syndrome. The Lancet.

[102] Wauters L, Talley NJ, Walker MM, Tack J, Vanuytsel T (2020). Novel concepts in the pathophysiology and treatment of functional dyspepsia. Gut.

[103] Mearin F, Malfertheiner P (2017). Functional gastrointestinal disorders: complex treatments for complex pathophysiological mechanisms. Dig Dis.

[104] Van Oudenhove L, Demyttenaere K, Tack J, Aziz Q (2004). Central nervous system involvement in functional gastrointestinal disorders. Best Pract Res Clin Gastroenterol.

[105] Sperber AD, Bangdiwala SI, Drossman DA, Ghoshal UC, Simren M, Tack J, Whitehead WE, Dumitrascu DL, Fang X, Fukudo S (2020). Worldwide prevalence and burden of functional gastrointestinal disorders, results of Rome Foundation Global Study. Gastroenterology.

[106] Tack J, Stanghellini V, Mearin F, Yiannakou Y, Layer P, Coffin B, Simren M, Mackinnon J, Wiseman G, Marciniak A (2019). Economic burden of moderate to severe irritable bowel syndrome with constipation in six European countries. BMC Gastroenterol.

[107] Dothel G, Barbaro MR, Boudin H, Vasina V, Cremon C, Gargano L, Bellacosa L, De Giorgio R, Le Berre-Scoul C, Aubert P, Neunlist M, De Ponti F, Stanghellini V, Barbara G (2015). Nerve fiber outgrowth is increased in the intestinal mucosa of patients with irritable bowel syndrome. Gastroenterology.

[108] Wood JD, Liu S, Drossman DA, Ringel Y, Whitehead WE (2012). Anti-enteric neuronal antibodies and the irritable bowel syndrome. J Neurogastroenterol Motil.

[109] Törnblom H, Lindberg G, Nyberg B, Veress B (2002). Full-thickness biopsy of the jejunum reveals inflammation and enteric neuropathy in irritable bowel syndrome. Gastroenterology.

[110] Tornblom H, Lang B, Clover L, Knowles CH, Vincent A, Lindberg G (2007). Autoantibodies in patients with gut motility disorders and enteric neuropathy. Scand J Gastroenterol.

[111] Cirillo C, Bessissow T, Desmet AS, Vanheel H, Tack J, Vanden Berghe P (2015). Evidence for neuronal and structural changes in submucous ganglia of patients with functional dyspepsia. Am J Gastroenterol.

[112] Sasselli V, Boesmans W, Vanden Berghe P, Tissir F, Goffinet AM, Pachnis V (2013). Planar cell polarity genes control the connectivity of enteric neurons. J Clin Invest.

[113] Gazouli M, Wouters MM, Kapur-Pojskić L, Bengtson MB, Friedman E, Nikčević G, Demetriou CA, Mulak A, Santos J, Niesler B (2016). Lessons learned–resolving the enigma of genetic factors in IBS. Nat Rev Gastroenterol Hepatol.

[114] Wouters MM, Lambrechts D, Knapp M, Cleynen I, Whorwell P, Agréus L, Dlugosz A, Schmidt PT, Halfvarson J, Simrén M, Ohlsson B, Karling P, Van Wanrooy S, Mondelaers S, Vermeire S, Lindberg G, Spiller R, Dukes G, D'Amato M, Boeckxstaens G (2014). Genetic variants in CDC42 and NXPH1 as susceptibility factors for constipation and diarrhoea predominant irritable bowel syndrome. Gut.

[115] Wohlfarth C, Schmitteckert S, Härtle JD, Houghton LA, Dweep H, Fortea M, Assadi G, Braun A, Mederer T, Pöhner S, Becker PP, Fischer C, Granzow M, Mönnikes H, Mayer EA, Sayuk G, Boeckxstaens G, Wouters MM, Simrén M, Lindberg G, Ohlsson B, Schmidt PT, Dlugosz A, Agreus L, Andreasson A, D'Amato M, Burwinkel B, Bermejo JL, Röth R, Lasitschka F, Vicario M, Metzger M, Santos J, Rappold GA, Martinez C, Niesler B (2017). miR-16 and miR-103 impact 5-HT(4) receptor signalling and correlate with symptom profile in irritable bowel syndrome. Sci Rep.

[116] Niesler B, Hattensperger N, Martinez C, Schmitteckert S, Houghton LA, Goebel-Stengel M, Knab D, Hammer C, D'Amato M, Zheng T, Moennikes H, Berens S, Kraus F, Andresen V, Frieling T, Keller J, Pehl C, Thoeringer C, Hoffmann P, Noethen MM, Heilmann-Heimbach S, Franke A, Lieb W, Clarke G, Cryan JF, Dinan TG, Quigley EM, Spiller R, Beltran C, Herzog W, Vicario M, Santos J, Mayer EA, Sayuk G, Gazouli M, Bustamante M, Rabionet K, Estivill X, Boeckxstaens G, Wouters MM, Simren M, Kabisch M, Raithel M, Rappold GA, Schaefert R, Lorenzo-Bermejo J (2018) The Serotonin receptor 3E subunit variant HTR3E c.*76G> A is confirmed as a risk factor for IBS-D in females. Neurogastroenterol Motil 30(S1):e13422

[117] Kapeller J, Houghton LA, Mönnikes H, Walstab J, Möller D, Bönisch H, Burwinkel B, Autschbach F, Funke B, Lasitschka F, Gassler N, Fischer C, Whorwell PJ, Atkinson W, Fell C, Büchner KJ, Schmidtmann M, van der Voort I, Wisser AS, Berg T, Rappold G, Niesler B (2008). First evidence for an association of a functional variant in the microRNA-510 target site of the serotonin receptor-type 3E gene with diarrhea predominant irritable bowel syndrome. Hum Mol Genet.

[118] Kumar S, Ranjan P, Mittal B, Ghoshal UC (2012). Serotonin transporter gene (SLC6A4) polymorphism in patients with irritable bowel syndrome and healthy controls. J Gastrointestin Liver Dis.

[119] Niesler B, Kapeller J, Fell C, Atkinson W, Möller D, Fischer C, Whorwell P, Houghton LA (2010). 5-HTTLPR and STin2 polymorphisms in the serotonin transporter gene and irritable bowel syndrome: effect of bowel habit and sex. Eur J Gastroenterol Hepatol.

[120] Ek WE, Reznichenko A, Ripke S, Niesler B, Zucchelli M, Rivera NV, Schmidt PT, Pedersen NL, Magnusson P, Talley NJ, Holliday EG, Houghton L, Gazouli M, Karamanolis G, Rappold G, Burwinkel B, Surowy H, Rafter J, Assadi G, Li L, Papadaki E, Gambaccini D, Marchi S, Colucci R, Blandizzi C, Barbaro R, Karling P, Walter S, Ohlsson B, Tornblom H, Bresso F, Andreasson A, Dlugosz A, Simren M, Agreus L, Lindberg G, Boeckxstaens G, Bellini M, Stanghellini V, Barbara G, Daly MJ, Camilleri M, Wouters MM, Amato M (2015). Exploring the genetics of irritable bowel syndrome: a GWA study in the general population and replication in multinational case-control cohorts. Gut.

[121] Holliday EG, Attia J, Hancock S, Koloski N, McEvoy M, Peel R, D'Amato M, Agréus L, Nyhlin H, Andreasson A, Almazar AE, Saito YA, Scott RJ, Talley NJ (2014). Genome-wide association study identifies two novel genomic regions in irritable bowel syndrome. Am J Gastroenterol.

[122] Bonfiglio F, Henström M, Nag A, Hadizadeh F, Zheng T, Cenit MC, Tigchelaar E, Williams F, Reznichenko A, Ek WE, Rivera NV, Homuth G, Aghdassi AA, Kacprowski T, Männikkö M, Karhunen V, Bujanda L, Rafter J, Wijmenga C, Ronkainen J, Hysi P, Zhernakova A, D'Amato M (2018). A GWAS meta-analysis from 5 population-based cohorts implicates ion channel genes in the pathogenesis of irritable bowel syndrome. Neurogastroenterol Motil.

[123] Hyland NP, Cryan JF (2016). Microbe-host interactions: Influence of the gut microbiota on the enteric nervous system. Dev Biol.

[124] Vincent AD, Wang X-Y, Parsons SP, Khan WI, Huizinga JD (2018). Abnormal absorptive colonic motor activity in germ-free mice is rectified by butyrate, an effect possibly mediated by mucosal serotonin. Am J Physiol-Gastrointestinal Liver Physiol.

[125] Ge X, Ding C, Zhao W, Xu L, Tian H, Gong J, Zhu M, Li J, Li N (2017). Antibiotics-induced depletion of mice microbiota induces changes in host serotonin biosynthesis and intestinal motility. J Transl Med.

[126] McVey Neufeld K, Mao Y, Bienenstock J, Foster J, Kunze W (2013). The microbiome is essential for normal gut intrinsic primary afferent neuron excitability in the mouse. Neurogastroenterol Motil.

[127] Furness J, Stebbing M (2018). The first brain: species comparisons and evolutionary implications for the enteric and central nervous systems. Neurogastroenterol Motil.

[128] Obata Y, Pachnis V (2020). Linking neurons to immunity: Lessons from Hydra. Proc Natl Acad Sci USA.

[129] Murillo-Rincon AP, Klimovich A, Pemöller E, Taubenheim J, Mortzfeld B, Augustin R, Bosch TC (2017). Spontaneous body contractions are modulated by the microbiome of Hydra. Sci Rep.

[130] Campbell R, Josephson R, Schwab W, Rushforth N (1976). Excitability of nerve-free hydra. Nature.

[131] Klimovich A, Giacomello S, Björklund Å, Faure L, Kaucka M, Giez C, Murillo-Rincon AP, Matt A-S, Willoweit-Ohl D, Crupi G (2020). Prototypical pacemaker neurons interact with the resident microbiota. Proc Natl Acad Sci.

[132] Pimentel M, Lembo A (2020). Microbiome and its role in irritable bowel syndrome. Dig Dis Sci.

[133] Tziatzios G, Gkolfakis P, Papanikolaou IS, Mathur R, Pimentel M, Giamarellos-Bourboulis EJ, Triantafyllou K (2020). Gut microbiota dysbiosis in functional dyspepsia. Microorganisms.

[134] Jankipersadsing SA, Hadizadeh F, Bonder MJ, Tigchelaar EF, Deelen P, Fu J, Andreasson A, Agreus L, Walter S, Wijmenga C, Hysi P, D'Amato M, Zhernakova A (2017). A GWAS meta-analysis suggests roles for xenobiotic metabolism and ion channel activity in the biology of stool frequency. Gut.

[135] Clarke G, McKernan D, Gaszner G, Quigley E, Cryan J, Dinan T (2012). A distinct profile of tryptophan metabolism along the kynurenine pathway downstream of toll-like receptor activation in irritable bowel syndrome. Front Pharmacol.

[136] Zelante T, Iannitti RG, Cunha C, De Luca A, Giovannini G, Pieraccini G, Zecchi R, D’Angelo C, Massi-Benedetti C, Fallarino F (2013). Tryptophan catabolites from microbiota engage aryl hydrocarbon receptor and balance mucosal reactivity via interleukin-22. Immunity.

[137] Dunlop SP, Jenkins D, Neal KR, Spiller RC (2003). Relative importance of enterochromaffin cell hyperplasia, anxiety, and depression in postinfectious IBS. Gastroenterology.

[138] Spiller RC, Jenkins D, Thornley JP, Hebden JM, Wright T, Skinner M, Neal KR (2000). Increased rectal mucosal enteroendocrine cells, T lymphocytes, and increased gut permeability following acute Campylobacter enteritis and in post-dysenteric irritable bowel syndrome. Gut.

[139] Yano JM, Yu K, Donaldson GP, Shastri GG, Ann P, Ma L, Nagler CR, Ismagilov RF, Mazmanian SK, Hsiao EY (2015). Indigenous bacteria from the gut microbiota regulate host serotonin biosynthesis. Cell.

[140] Talley NJ, Cook DR (2019) Functional dyspepsia. In: Essential medical disorders of the stomach and small intestine. Springer, Berlin, pp 155–172

[141] Barbara G, Grover M, Bercik P, Corsetti M, Ghoshal UC, Ohman L, Rajilić-Stojanović M (2019). Rome Foundation working team report on post-infection irritable bowel syndrome. Gastroenterology.

[142] Balemans D, Mondelaers S, Cibert-Goton V, Stakenborg N, Aguilera-Lizarraga J, Dooley J, Liston A, Bulmer D, Berghe PV, Boeckxstaens G (2017). Evidence for long-term sensitization of the bowel in patients with post-infectious-IBS. Sci Rep.

[143] Matheis F, Muller PA, Graves CL, Gabanyi I, Kerner ZJ, Costa-Borges D, Ahrends T, Rosenstiel P, Mucida D (2020). Adrenergic signaling in muscularis macrophages limits infection-induced neuronal loss. Cell.

[144] Matteoli G, Gomez-Pinilla PJ, Nemethova A, Di Giovangiulio M, Cailotto C, van Bree SH, Michel K, Tracey KJ, Schemann M, Boesmans W, Vanden Berghe P, Boeckxstaens GE (2014). A distinct vagal anti-inflammatory pathway modulates intestinal muscularis resident macrophages independent of the spleen. Gut.

[145] De Schepper S, Verheijden S, Aguilera-Lizarraga J, Viola MF, Boesmans W, Stakenborg N, Voytyuk I, Schmidt I, Boeckx B, Dierckx de Casterle I, Baekelandt V, Gonzalez Dominguez E, Mack M, Depoortere I, De Strooper B, Sprangers B, Himmelreich U, Soenen S, Guilliams M, Vanden Berghe P, Jones E, Lambrechts D, Boeckxstaens G (2018). Self-maintaining gut macrophages are essential for intestinal homeostasis. Cell.

[146] Muller PA, Koscso B, Rajani GM, Stevanovic K, Berres ML, Hashimoto D, Mortha A, Leboeuf M, Li XM, Mucida D, Stanley ER, Dahan S, Margolis KG, Gershon MD, Merad M, Bogunovic M (2014). Crosstalk between muscularis macrophages and enteric neurons regulates gastrointestinal motility. Cell.

[147] Rodríguez-Fandiño OA, Hernández-Ruiz J, López-Vidal Y, Charúa-Guindic L, Escobedo G, Schmulson MJ (2017). Maturation phenotype of peripheral blood monocyte/macrophage after stimulation with lipopolysaccharides in irritable bowel syndrome. J Neurogastroenterol Motil.

[148] Boyer J, Saint-Paul M-C, Dadone B, Patouraux S, Vivinus M-H, Ouvrier D, Michiels J-F, Piche T, Tulic MK (2018). Inflammatory cell distribution in colon mucosa as a new tool for diagnosis of irritable bowel syndrome: a promising pilot study. Neurogastroenterol Motil.

[149] Pixley FJ, Stanley ER (2004). CSF-1 regulation of the wandering macrophage: complexity in action. Trends Cell Biol.

[150] Grubišić V, McClain JL, Fried DE, Grants I, Rajasekhar P, Csizmadia E, Ajijola OA, Watson RE, Poole DP, Robson SC (2020). Enteric glia modulate macrophage phenotype and visceral sensitivity following inflammation. Cell Rep.

[151] Avetisyan M, Rood JE, Lopez SH, Sengupta R, Wright-Jin E, Dougherty JD, Behrens EM, Heuckeroth RO (2018). Muscularis macrophage development in the absence of an enteric nervous system. Proc Natl Acad Sci.

[152] van Wanrooij SJ, Wouters MM, Van Oudenhove L, Vanbrabant W, Mondelaers S, Kollmann P, Kreutz F, Schemann M, Boeckxstaens GE (2014). Sensitivity testing in irritable bowel syndrome with rectal capsaicin stimulations: role of TRPV1 upregulation and sensitization in visceral hypersensitivity?. Am J Gastroenterol.

[153] Aguilera-Lizarraga J, Florens MV, Viola MF, Jain P, Decraecker L, Appeltans I, Cuende-Estevez M, Fabre N, Van Beek K, Perna E, Balemans D, Stakenborg N, Theofanous S, Bosmans G, Mondelaers SU, Matteoli G, Ibiza Martínez S, Lopez-Lopez C, Jaramillo-Polanco J, Talavera K, Alpizar YA, Feyerabend TB, Rodewald H-R, Farre R, Redegeld FA, Si J, Raes J, Breynaert C, Schrijvers R, Bosteels C, Lambrecht BN, Boyd SD, Hoh RA, Cabooter D, Nelis M, Augustijns P, Hendrix S, Strid J, Bisschops R, Reed DE, Vanner SJ, Denadai-Souza A, Wouters MM, Boeckxstaens GE (2021). Local immune response to food antigens drives meal-induced abdominal pain. Nature.

[154] Jimenez-Vargas NN, Pattison LA, Zhao P, Lieu T, Latorre R, Jensen DD, Castro J, Aurelio L, Le GT, Flynn B, Herenbrink CK, Yeatman HR, Edgington-Mitchell L, Porter CJH, Halls ML, Canals M, Veldhuis NA, Poole DP, McLean P, Hicks GA, Scheff N, Chen E, Bhattacharya A, Schmidt BL, Brierley SM, Vanner SJ, Bunnett NW (2018). Protease-activated receptor-2 in endosomes signals persistent pain of irritable bowel syndrome. Proc Natl Acad Sci USA.

[155] Grundy L, Erickson A, Brierley SM (2019). Visceral pain. Annu Rev Physiol.

[156] Castro J, Harrington AM, Lieu T, Garcia-Caraballo S, Maddern J, Schober G, O'Donnell T, Grundy L, Lumsden AL, Miller P, Ghetti A, Steinhoff MS, Poole DP, Dong X, Chang L, Bunnett NW, Brierley SM (2019). Activation of pruritogenic TGR5, MrgprA3, and MrgprC11 on colon-innervating afferents induces visceral hypersensitivity. JCI Insight.

[157] Sessenwein JL, Baker CC, Pradhananga S, Maitland ME, Petrof EO, Allen-Vercoe E, Noordhof C, Reed DE, Vanner SJ, Lomax AE (2017). Protease-mediated suppression of DRG neuron excitability by commensal bacteria. J Neurosci.

[158] Hughes PA, Brierley SM, Martin CM, Brookes SJ, Linden DR, Blackshaw LA (2009). Post-inflammatory colonic afferent sensitisation: different subtypes, different pathways and different time courses. Gut.

[159] Desormeaux C, Bautzova T, Garcia-Caraballo S, Rolland C, Barbaro MR, Brierley SM, Barbara G, Vergnolle N, Cenac N (2018). Protease-activated receptor 1 is implicated in irritable bowel syndrome mediators-induced signaling to thoracic human sensory neurons. Pain.

[160] Corsetti M, Akyuz F, Tack J (2015). Targeting tachykinin receptors for the treatment of functional gastrointestinal disorders with a focus on irritable bowel syndrome. Neurogastroenterol Motil.

[161] Cenac N, Bautzova T, Le Faouder P, Veldhuis NA, Poole DP, Rolland C, Bertrand J, Liedtke W, Dubourdeau M, Bertrand-Michel J (2015). Quantification and potential functions of endogenous agonists of transient receptor potential channels in patients with irritable bowel syndrome. Gastroenterology.

[162] O’sullivan M, Clayton N, Breslin N, Harman I, Bountra C, McLaren A, O’Morain C (2000). Increased mast cells in the irritable bowel syndrome. Neurogastroenterol Motil.

[163] Vanheel H, Farré R (2013). Changes in gastrointestinal tract function and structure in functional dyspepsia. Nat Rev Gastroenterol Hepatol.

[164] Vanheel H, Vicario M, Boesmans W, Vanuytsel T, Salvo-Romero E, Tack J, Farre R (2018). Activation of eosinophils and mast cells in functional dyspepsia: an ultrastructural evaluation. Sci Rep.

[165] Philpott H, Gibson P, Thien F (2011). Irritable bowel syndrome-An inflammatory disease involving mast cells. Asia Pac Allergy.

[166] Liang W-J, Zhang G, Luo H-S, Liang L-X, Huang D, Zhang F-C (2016). Tryptase and protease-activated receptor 2 expression levels in irritable bowel syndrome. Gut Liver.

[167] Martínez C, Lasitschka F, Thöni C, Wohlfarth C, Braun A, Granzow M, Röth R, Dizdar V, Rappold GA, Hausken T (2020). Comparative expression profiling in the intestine of patients with Giardia-induced postinfectious functional gastrointestinal disorders. Neurogastroenterol Motil.

[168] Xu XJ, Zhang YL, Liu L, Pan L, Yao SK (2017). Increased expression of nerve growth factor correlates with visceral hypersensitivity and impaired gut barrier function in diarrhoea-predominant irritable bowel syndrome: a preliminary explorative study. Aliment Pharmacol Ther.

[169] He SH, He YS, Xie H (2004). Activation of human colon mast cells through proteinase activated receptor-2. World J Gastroenterol.

[170] Barbara G, Stanghellini V, De Giorgio R, Cremon C, Cottrell GS, Santini D, Pasquinelli G, Morselli-Labate AM, Grady EF, Bunnett NW (2004). Activated mast cells in proximity to colonic nerves correlate with abdominal pain in irritable bowel syndrome. Gastroenterology.

[171] Palsson OS, Morteau O, Bozymski EM, Woosley JT, Sartor RB, Davies MJ, Johnson DA, Turner MJ, Whitehead WE (2004). Elevated vasoactive intestinal peptide concentrations in patients with irritable bowel syndrome. Dig Dis Sci.

[172] Rosa AC, Fantozzi R (2013). The role of histamine in neurogenic inflammation. Br J Pharmacol.

[173] Buhner S, Barki N, Greiter W, Giesbertz P, Demir IE, Ceyhan GO, Zeller F, Daniel H, Schemann M (2017). Calcium imaging of nerve-mast cell signaling in the human intestine. Front Physiol.

[174] Buhner S, Li Q, Vignali S, Barbara G, De Giorgio R, Stanghellini V, Cremon C, Zeller F, Langer R, Daniel H (2009). Activation of human enteric neurons by supernatants of colonic biopsy specimens from patients with irritable bowel syndrome. Gastroenterology.

[175] Buhner S, Li Q, Berger T, Vignali S, Barbara G, De Giorgio R, Stanghellini V, Schemann M (2012). Submucous rather than myenteric neurons are activated by mucosal biopsy supernatants from irritable bowel syndrome patients. Neurogastroenterol Motil.

[176] Buhner S, Braak B, Li Q, Kugler EM, Klooker T, Wouters M, Donovan J, Vignali S, Mazzuoli-Weber G, Grundy D (2014). Neuronal activation by mucosal biopsy supernatants from irritable bowel syndrome patients is linked to visceral sensitivity. Exp Physiol.

[177] Barbara G, Wang B, Stanghellini V, De Giorgio R, Cremon C, Di Nardo G, Trevisani M, Campi B, Geppetti P, Tonini M (2007). Mast cell-dependent excitation of visceral-nociceptive sensory neurons in irritable bowel syndrome. Gastroenterology.

[178] Cenac N, Andrews CN, Holzhausen M, Chapman K, Cottrell G, Andrade-Gordon P, Steinhoff M, Barbara G, Beck P, Bunnett NW (2007). Role for protease activity in visceral pain in irritable bowel syndrome. J Clin Investig.

[179] Valdez-Morales EE, Overington J, Guerrero-Alba R, Ochoa-Cortes F, Ibeakanma CO, Spreadbury I, Bunnett NW, Beyak M, Vanner SJ (2013). Sensitization of peripheral sensory nerves by mediators from colonic biopsies of diarrhea-predominant irritable bowel syndrome patients: a role for PAR2. Am J Gastroenterol.

[180] Wouters MM, Vicario M, Santos J (2016). The role of mast cells in functional GI disorders. Gut.

[181] Scanzi J, Accarie A, Muller E, Pereira B, Aissouni Y, Goutte M, Joubert-Zakeyh J, Picard E, Mallet C, Gelot A (2016). Colonic overexpression of the T-type calcium channel Cav32 in a mouse model of visceral hypersensitivity and in irritable bowel syndrome patients. Neurogastroenterol Motil.

[182] Akbar A, Yiangou Y, Facer P, Walters JR, Anand P, Ghosh S (2008). Increased capsaicin receptor TRPV1-expressing sensory fibres in irritable bowel syndrome and their correlation with abdominal pain. Gut.

[183] Akbar A, Yiangou Y, Facer P, Brydon W, Walters JR, Anand P, Ghosh S (2010). Expression of the TRPV1 receptor differs in quiescent inflammatory bowel disease with or without abdominal pain. Gut.

[184] Holzer P (2008). TRPV1: a new target for treatment of visceral pain in IBS?. Gut.

[185] Boesmans W, Owsianik G, Tack J, Voets T, Vanden Berghe P (2011). TRP channels in neurogastroenterology: opportunities for therapeutic intervention. Br J Pharmacol.

[186] Buckinx R, Van Nassauw L, Avula LR, Alpaerts K, Adriaensen D, Timmermans JP (2013). Transient receptor potential vanilloid type 1 channel (TRPV1) immunolocalization in the murine enteric nervous system is affected by the targeted C-terminal epitope of the applied antibody. J Histochem Cytochem.

[187] Ward SM, Bayguinov J, Won KJ, Grundy D, Berthoud HR (2003). Distribution of the vanilloid receptor (VR1) in the gastrointestinal tract. J Comp Neurol.

[188] Sharrad DF, Hibberd TJ, Kyloh MA, Brookes SJ, Spencer NJ (2015). Quantitative immunohistochemical co-localization of TRPV1 and CGRP in varicose axons of the murine oesophagus, stomach and colorectum. Neurosci Lett.

[189] Spencer NJ, Magnúsdóttir EI, Jakobsson JET, Kestell G, Chen BN, Morris D, Brookes SJ, Lagerström MC (2017). CGRPα within the Trpv1-Cre population contributes to visceral nociception. Am J Physiol-Gastr L.

[190] Delvalle NM, Dharshika C, Morales-Soto W, Fried DE, Gaudette L, Gulbransen BD (2018). Communication between enteric neurons, glia, and nociceptors underlies the effects of tachykinins on neuroinflammation. Cell Mol Gastroenterol Hepatol.

[191] Wouters MM, Balemans D, Van Wanrooy S, Dooley J, Cibert-Goton V, Alpizar YA, Valdez-Morales EE, Nasser Y, Van Veldhoven PP, Vanbrabant W (2016). Histamine receptor H1–mediated sensitization of TRPV1 mediates visceral hypersensitivity and symptoms in patients with irritable bowel syndrome. Gastroenterology.

[192] Lilli NL, Quénéhervé L, Haddara S, Brochard C, Aubert P, Rolli-Derkinderen M, Durand T, Naveilhan P, Hardouin JB, De Giorgio R, Barbara G, des Varannes SB, Coron E, Neunlist M (2018). Glioplasticity in irritable bowel syndrome. Neurogastroenterol Motil.

[193] Steinhoff MS, von Mentzer B, Geppetti P, Pothoulakis C, Bunnett NW (2014). Tachykinins and their receptors: contributions to physiological control and the mechanisms of disease. Physiol Rev.

[194] O'Connor TM, O'Connell J, O'Brien DI, Goode T, Bredin CP, Shanahan F (2004). The role of substance P in inflammatory disease. J Cell Physiol.

[195] Carini F, Lecci A, Tramontana M, Giuliani S, Maggi C (2001). Tachykinin NK2 receptors and enhancement of cholinergic transmission in the inflamed rat colon: an in vivo motility study. Br J Pharmacol.

[196] Patel BA, Patel N, Fidalgo S, Wang C, Ranson RN, Saffrey MJ, Yeoman MS (2014). Impaired colonic motility and reduction in tachykinin signalling in the aged mouse. Exp Gerontol.

[197] Szymaszkiewicz A, Malkiewicz A, Storr M, Fichna J, Zielinska M (2019). The place of tachykinin NK2 receptor antagonists in the treatment diarrhea-predominant irritable bowel syndrome. J Physiol Pharmacol.

[198] Mars RAT, Yang Y, Ward T, Houtti M, Priya S, Lekatz HR, Tang X, Sun Z, Kalari KR, Korem T, Bhattarai Y, Zheng T, Bar N, Frost G, Johnson AJ, van Treuren W, Han S, Ordog T, Grover M, Sonnenburg J, D’Amato M, Camilleri M, Elinav E, Segal E, Blekhman R, Farrugia G, Swann JR, Knights D, Kashyap PC (2020). Longitudinal multi-omics reveals subset-specific mechanisms underlying irritable bowel syndrome. Cell.

[199] Simren M, Tornblom H, Palsson OS, Van Oudenhove L, Whitehead WE, Tack J (2019). Cumulative effects of psychologic distress, visceral hypersensitivity, and abnormal transit on patient-reported outcomes in irritable bowel syndrome. Gastroenterology.

[200] Bonfiglio F, Zheng T, Garcia-Etxebarria K, Hadizadeh F, Bujanda L, Bresso F, Agreus L, Andreasson A, Dlugosz A, Lindberg G, Schmidt PT, Karling P, Ohlsson B, Simren M, Walter S, Nardone G, Cuomo R, Usai-Satta P, Galeazzi F, Neri M, Portincasa P, Bellini M, Barbara G, Latiano A, Hübenthal M, Thijs V, Netea MG, Jonkers D, Chang L, Mayer EA, Wouters MM, Boeckxstaens G, Camilleri M, Franke A, Zhernakova A, D’Amato M (2018). Female-specific association between variants on chromosome 9 and self-reported diagnosis of irritable bowel syndrome. Gastroenterology.

[201] Zhao L, Yang W, Chen Y, Huang F, Lu L, Lin C, Huang T, Ning Z, Zhai L, Zhong LL, Lam W, Yang Z, Zhang X, Cheng C, Han L, Qiu Q, Shang X, Huang R, Xiao H, Ren Z, Chen D, Sun S, El-Nezami H, Cai Z, Lu A, Fang X, Jia W, Bian Z (2020). A Clostridia-rich microbiota enhances bile acid excretion in diarrhea-predominant irritable bowel syndrome. J Clin Investig.

[202] Jairath V, Feagan BG (2020). Global burden of inflammatory bowel disease. Lancet Gastroenterol Hepatol.

[203] Neurath MF (2019). Targeting immune cell circuits and trafficking in inflammatory bowel disease. Nat Immunol.

[204] Neurath MF (2020). Host-microbiota interactions in inflammatory bowel disease. Nat Rev Gastroenterol Hepatol.

[205] Ferrante M, De Hertogh G, Hlavaty T, D’Haens G, Penninckx F, D’Hoore A, Vermeire S, Rutgeerts P, Geboes K, Van Assche G (2006). The value of myenteric plexitis to predict early postoperative Crohn’s disease recurrence. Gastroenterology.

[206] Villanacci V, Bassotti G, Nascimbeni R, Antonelli E, Cadei M, Fisogni S, Salerni B, Geboes K (2008). Enteric nervous system abnormalities in inflammatory bowel diseases. Neurogastroenterol Motil.

[207] Sokol H, Polin V, Lavergne-Slove A, Panis Y, Treton X, Dray X, Bouhnik Y, Valleur P, Marteau P (2009). Plexitis as a predictive factor of early postoperative clinical recurrence in Crohn’s disease. Gut.

[208] Lakhan SE, Kirchgessner A (2010). Neuroinflammation in inflammatory bowel disease. J Neuroinflammation.

[209] Geboes K, Collins S (1998). Structural abnormalities of the nervous system in Crohn's disease and ulcerative colitis. Neurogastroenterol Motil.

[210] Brierley SM, Linden DR (2014). Neuroplasticity and dysfunction after gastrointestinal inflammation. Nat Rev Gastroenterol Hepatol.

[211] Linden DR, Sharkey KA, Mawe GM (2003). Enhanced excitability of myenteric AH neurones in the inflamed guinea-pig distal colon. J Physiol.

[212] Lomax AE, Mawe GM, Sharkey KA (2005). Synaptic facilitation and enhanced neuronal excitability in the submucosal plexus during experimental colitis in guinea-pig. J Physiol.

[213] Krauter EM, Linden DR, Sharkey KA, Mawe GM (2007). Synaptic plasticity in myenteric neurons of the guinea-pig distal colon: presynaptic mechanisms of inflammation-induced synaptic facilitation. J Physiol.

[214] Mizuta Y, Isomoto H, Takahashi T (2000). Impaired nitrergic innervation in rat colitis induced by dextran sulfate sodium. Gastroenterology.

[215] Depoortere I, Thijs T, Peeters TL (2002). Generalized loss of inhibitory innervation reverses serotonergic inhibition into excitation in a rabbit model of TNBS-colitis. Br J Pharmacol.

[216] Strong DS, Cornbrooks CF, Roberts JA, Hoffman JM, Sharkey KA, Mawe GM (2010). Purinergic neuromuscular transmission is selectively attenuated in ulcerated regions of inflamed guinea pig distal colon. J Physiol.

[217] Gulbransen BD, Bashashati M, Hirota SA, Gui X, Roberts JA, MacDonald JA, Muruve DA, McKay DM, Beck PL, Mawe GM (2012). Activation of neuronal P2X7 receptor–pannexin-1 mediates death of enteric neurons during colitis. Nat Med.

[218] Mawe GM (2015). Colitis-induced neuroplasticity disrupts motility in the inflamed and post-inflamed colon. J Clin Invest.

[219] Margolis KG, Stevanovic K, Karamooz N, Li ZS, Ahuja A, D'Autréaux F, Saurman V, Chalazonitis A, Gershon MD (2011). Enteric neuronal density contributes to the severity of intestinal inflammation. Gastroenterology.

[220] Pacheco R, Contreras F, Prado C (2012). Cells, molecules and mechanisms involved in the neuro-immune interaction. Cell Interact.

[221] Kioussis D, Pachnis V (2009). Immune and nervous systems: more than just a superficial similarity?. Immunity.

[222] Godinho-Silva C, Cardoso F, Veiga-Fernandes H (2019). Neuro–immune cell units: a new paradigm in physiology. Annu Rev Immunol.

[223] Huh JR, Veiga-Fernandes H (2019). Neuroimmune circuits in inter-organ communication. Nature Rev Immunol.

[224] Nowarski R, Jackson R, Gagliani N, de Zoete MR, Palm NW, Bailis W, Low JS, Harman CC, Graham M, Elinav E, Flavell RA (2015). Epithelial IL-18 equilibrium controls barrier function in colitis. Cell.

[225] Jarret A, Jackson R, Duizer C, Healy ME, Zhao J, Rone JM, Bielecki P, Sefik E, Roulis M, Rice T, Sivanathan KN, Zhou T, Solis AG, Honcharova-Biletska H, Vélez K, Hartner S, Low JS, Qu R, de Zoete MR, Palm NW, Ring AM, Weber A, Moor AE, Kluger Y, Nowarski R, Flavell RA (2020). Enteric nervous system-derived IL-18 orchestrates mucosal barrier immunity. Cell.

[226] Williams MA, O'Callaghan A, Corr SC (2019). IL-33 and IL-18 in inflammatory bowel disease etiology and microbial interactions. Front Immunol.

[227] Bank S, Andersen PS, Burisch J, Pedersen N, Roug S, Galsgaard J, Turino SY, Brodersen JB, Rashid S, Rasmussen BK, Avlund S, Olesen TB, Hoffmann HJ, Nexø BA, Sode J, Vogel U, Andersen V (2018). Genetically determined high activity of IL-12 and IL-18 in ulcerative colitis and TLR5 in Crohns disease were associated with non-response to anti-TNF therapy. Pharmacogenomics J.

[228] Chu C, Artis D, Chiu IM (2020). Neuro-immune interactions in the tissues. Immunity.

[229] Mashaghi A, Marmalidou A, Tehrani M, Grace PM, Pothoulakis C, Dana R (2016). Neuropeptide substance P and the immune response. Cell Mol Life Sci.

[230] Neunlist M, Aubert P, Toquet C, Oreshkova T, Barouk J, Lehur P, Schemann M, Galmiche J (2003). Changes in chemical coding of myenteric neurones in ulcerative colitis. Gut.

[231] O’Connor T, O’Connell J, O’Brien DI, Goode T, Bredin CP, Shanahan F (2004). The role of substance P in inflammatory disease. J Cell Physiol.

[232] Schneider J, Jehle E, Starlinger M, Neunlist M, Michel K, Hoppe S, Schemann M (2001). Neurotransmitter coding of enteric neurones in the submucous plexus is changed in non-inflamed rectum of patients with Crohn’s disease. Neurogastroenterol Motil.

[233] Boyer L, Sidpra D, Jevon G, Buchan AM, Jacobson K (2007). Differential responses of VIPergic and nitrergic neurons in paediatric patients with Crohn's disease. Auton Neurosci.

[234] Stead RH, Dixon MF, Bramwell NH, Riddell RH, Bienenstock J (1989). Mast cells are closely apposed to nerves in the human gastrointestinal mucosa. Gastroenterology.

[235] Kulka M, Sheen CH, Tancowny BP, Grammer LC, Schleimer RP (2008). Neuropeptides activate human mast cell degranulation and chemokine production. Immunology.

[236] Tamura K, Wood JD (1992). Effects of prolonged exposure to histamine on guinea pig intestinal neurons. Dig Dis Sci.

[237] Reed DE, Barajas-Lopez C, Cottrell G, Velazquez-Rocha S, Dery O, Grady EF, Bunnett NW, Vanner SJ (2003). Mast cell tryptase and proteinase-activated receptor 2 induce hyperexcitability of guinea-pig submucosal neurons. J Physiol.

[238] Nguyen C, Coelho A-M, Grady E, Compton SJ, Wallace JL, Hollenberg MD, Cenac N, Garcia-Villar R, Bueno L, Steinhoff M (2003). Colitis induced by proteinase-activated receptor-2 agonists is mediated by a neurogenic mechanism. Can J Physiol Pharmacol.

[239] Raithel M, Winterkamp S, Pacurar A, Ulrich P, Hochberger J, Hahn E (2001). Release of mast cell tryptase from human colorectal mucosa in inflammatory bowel disease. Scand J Gastroenterol.

[240] Fox CC, Lazenby AJ, Moore WC, Yardley JH, Bayless TM, Lichtenstein LM (1990). Enhancement of human intestinal mast cell mediator release in active ulcerative colitis. Gastroenterology.

[241] He S-H (2004). Key role of mast cells and their major secretory products in inflammatory bowel disease. World J Gastroenterol.

[242] Casado-Bedmar M, Heil SD, Myrelid P, Söderholm JD, Keita ÅV (2019). Upregulation of intestinal mucosal mast cells expressing VPAC1 in close proximity to vasoactive intestinal polypeptide in inflammatory bowel disease and murine colitis. Neurogastroenterol Motil.

[243] Seillet C, Luong K, Tellier J, Jacquelot N, Shen RD, Hickey P, Wimmer VC, Whitehead L, Rogers K, Smyth GK (2019). The neuropeptide VIP confers anticipatory mucosal immunity by regulating ILC3 activity. Nature Immunol.

[244] Britanova L, Diefenbach A (2017). Interplay of innate lymphoid cells and the microbiota. Immunol Rev.

[245] Sonnenberg GF, Monticelli LA, Elloso MM, Fouser LA, Artis D (2011). CD4+ lymphoid tissue-inducer cells promote innate immunity in the gut. Immunity.

[246] Talbot J, Hahn P, Kroehling L, Nguyen H, Li D, Littman DR (2020). Feeding-dependent VIP neuron–ILC3 circuit regulates the intestinal barrier. Nature.

[247] Forkel M, Mjösberg J (2016). Dysregulation of group 3 innate lymphoid cells in the pathogenesis of inflammatory bowel disease. Curr Allergy Asthma Rep.

[248] Cardoso V, Chesné J, Ribeiro H, García-Cassani B, Carvalho T, Bouchery T, Shah K, Barbosa-Morais NL, Harris N, Veiga-Fernandes H (2017). Neuronal regulation of type 2 innate lymphoid cells via neuromedin U. Nature.

[249] Klose CS, Mahlakõiv T, Moeller JB, Rankin LC, Flamar A-L, Kabata H, Monticelli LA, Moriyama S, Putzel GG, Rakhilin N (2017). The neuropeptide neuromedin U stimulates innate lymphoid cells and type 2 inflammation. Nature.

[250] Martinez VG, O'Driscoll L (2015). Neuromedin U: a multifunctional neuropeptide with pleiotropic roles. Clin Chem.

[251] Ibiza S, García-Cassani B, Ribeiro H, Carvalho T, Almeida L, Marques R, Misic AM, Bartow-McKenney C, Larson DM, Pavan WJ (2016). Glial-cell-derived neuroregulators control type 3 innate lymphoid cells and gut defence. Nature.

[252] Steinkamp M, Gundel H, Schulte N, Spaniol U, Pflueger C, Zizer E, von Boyen GB (2012). GDNF protects enteric glia from apoptosis: evidence for an autocrine loop. BMC Gastroenterol.

[253] Zhang DK, He FQ, Li TK, Pang XH, Cui DJ, Xie Q, Huang XL, Gan HT (2010). Glial-derived neurotrophic factor regulates intestinal epithelial barrier function and inflammation and is therapeutic for murine colitis. J Pathol.

[254] von Boyen GB, Schulte N, Pflüger C, Spaniol U, Hartmann C, Steinkamp M (2011). Distribution of enteric glia and GDNF during gut inflammation. BMC Gastroenterol.

[255] Sofroniew MV (2009). Molecular dissection of reactive astrogliosis and glial scar formation. Trends Neurosci.

[256] Pochard C, Coquenlorge S, Jaulin J, Cenac N, Vergnolle N, Meurette G, Freyssinet M, Neunlist M, Rolli-Derkinderen M (2016). Defects in 15-HETE production and control of epithelial permeability by human enteric glial cells from patients with Crohn's disease. Gastroenterology.

[257] Savidge TC, Newman P, Pothoulakis C, Ruhl A, Neunlist M, Bourreille A, Hurst R, Sofroniew MV (2007). Enteric glia regulate intestinal barrier function and inflammation via release of S-nitrosoglutathione. Gastroenterology.

[258] Brown IA, McClain JL, Watson RE, Patel BA, Gulbransen BD (2016). Enteric glia mediate neuron death in colitis through purinergic pathways that require connexin-43 and nitric oxide. Cell Mol Gastroenterol Hepatol.

[259] Menchén L, Colón AL, Madrigal JL, Beltrán L, Botella S, Lizasoain I, Leza JC, Moro MA, Menchén P, Cos E (2004). Activity of inducible and neuronal nitric oxide synthases in colonic mucosa predicts progression of ulcerative colitis. Am J Gastroenterol.

[260] Cirillo C, Sarnelli G, Esposito G, Grosso M, Petruzzelli R, Izzo P, Cali G, Darmiento FP, Rocco A, Nardone G (2009). Increased mucosal nitric oxide production in ulcerative colitis is mediated in part by the enteroglial-derived S100B protein. Neurogastroenterol Motil.

[261] Costa DVS, Bon-Frauches AC, Silva A, Lima-Junior RCP, Martins CS, Leitao RFC, Freitas GB, Castelucci P, Bolick DT, Guerrant RL, Warren CA, Moura-Neto V, Brito GAC (2019). 5-Fluorouracil induces enteric neuron death and glial activation during intestinal mucositis via a S100B-RAGE-NFkappaB-dependent pathway. Sci Rep.

[262] Cario E (2010). Toll-like receptors in inflammatory bowel diseases: a decade later. Inflamm Bowel Dis.

[263] Lu Y, Li X, Liu S, Zhang Y, Zhang D (2018). Toll-like receptors and inflammatory bowel disease. Front Immunol.

[264] Brun P, Giron MC, Qesari M, Porzionato A, Caputi V, Zoppellaro C, Banzato S, Grillo AR, Spagnol L, De Caro R, Pizzuti D, Barbieri V, Rosato A, Sturniolo GC, Martines D, Zaninotto G, Palu G, Castagliuolo I (2013). Toll-like receptor 2 regulates intestinal inflammation by controlling integrity of the enteric nervous system. Gastroenterology.

[265] Esposito G, Capoccia E, Turco F, Palumbo I, Lu J, Steardo A, Cuomo R, Sarnelli G, Steardo L (2014). Palmitoylethanolamide improves colon inflammation through an enteric glia/toll like receptor 4-dependent PPAR-alpha activation. Gut.

[266] Fukata M, Chen A, Vamadevan AS, Cohen J, Breglio K, Krishnareddy S, Hsu D, Xu R, Harpaz N, Dannenberg AJ (2007). Toll-like receptor-4 promotes the development of colitis-associated colorectal tumors. Gastroenterology.

[267] Sánchez-Muñoz F, Fonseca-Camarillo G, Villeda-Ramírez MA, Miranda-Pérez E, Mendivil EJ, Barreto-Zúñiga R, Uribe M, Bojalil R, Domínguez-López A, Yamamoto-Furusho JK (2011). Transcript levels of Toll-Like Receptors 5, 8 and 9 correlate with inflammatory activity in Ulcerative Colitis. BMC Gastroenterol.

[268] Bank S, Skytt Andersen P, Burisch J, Pedersen N, Roug S, Galsgaard J, Ydegaard Turino S, Broder Brodersen J, Rashid S, Kaiser Rasmussen B, Avlund S, Bastholm Olesen T, Jürgen Hoffmann H, Kragh Thomsen M, Østergaard Thomsen V, Frydenberg M, Andersen Nexø B, Sode J, Vogel U, Andersen V (2014). Polymorphisms in the Inflammatory Pathway Genes TLR2, TLR4, TLR9, LY96, NFKBIA, NFKB1, TNFA, TNFRSF1A, IL6R, IL10, IL23R, PTPN22, and PPARG are associated with susceptibility of inflammatory bowel disease in a Danish Cohort. PLoS ONE.

[269] Cheng Y, Zhu Y, Huang X, Zhang W, Han Z, Liu S (2015). Association between TLR2 and TLR4 gene polymorphisms and the susceptibility to inflammatory bowel disease: a meta-analysis. PLoS ONE.

[270] Sharkey KA, Savidge TC (2014). Role of enteric neurotransmission in host defense and protection of the gastrointestinal tract. Auton Neurosci.

[271] Barajon I, Serrao G, Arnaboldi F, Opizzi E, Ripamonti G, Balsari A, Rumio C (2009). Toll-like receptors 3, 4, and 7 are expressed in the enteric nervous system and dorsal root ganglia. J Histochem Cytochem.

[272] Burgueño JF, Barba A, Eyre E, Romero C, Neunlist M, Fernández E (2016). TLR2 and TLR9 modulate enteric nervous system inflammatory responses to lipopolysaccharide. J Neuroinflammation.

[273] Caputi V, Marsilio I, Cerantola S, Roozfarakh M, Lante I, Galuppini F, Rugge M, Napoli E, Giulivi C, Orso G (2017). Toll-like receptor 4 modulates small intestine neuromuscular function through nitrergic and purinergic pathways. Front Pharmacol.

[274] Cerantola S, Caputi V, Marsilio I, Ridolfi M, Faggin S, Bistoletti M, Giaroni C, Giron MC (2020). Involvement of enteric glia in small intestine neuromuscular dysfunction of toll-like receptor 4-deficient mice. Cells.

[275] Smillie CS, Biton M, Ordovas-Montanes J, Sullivan KM, Burgin G, Graham DB, Herbst RH, Rogel N, Slyper M, Waldman J, Sud M, Andrews E, Velonias G, Haber AL, Jagadeesh K, Vickovic S, Yao J, Stevens C, Dionne D, Nguyen LT, Villani A-C, Hofree M, Creasey EA, Huang H, Rozenblatt-Rosen O, Garber JJ, Khalili H, Desch AN, Daly MJ, Ananthakrishnan AN, Shalek AK, Xavier RJ, Regev A (2019). Intra- and inter-cellular rewiring of the human colon during ulcerative colitis. Cell.

[276] Aballay A (2009). Neural regulation of immunity: role of NPR-1 in pathogen avoidance and regulation of innate immunity. Cell Cycle.

[277] Foster KJ, Cheesman HK, Liu P, Peterson ND, Anderson SM, Pukkila-Worley R (2020). Innate Immunity in the *C. elegans *intestine is programmed by a neuronal regulator of AWC Olfactory Neuron Development. Cell Rep.

[278] Veiga-Fernandes H, Pachnis V (2017). Neuroimmune regulation during intestinal development and homeostasis. Nat Immunol.

[279] Safiri S, Sepanlou SG, Ikuta KS, Bisignano C, Salimzadeh H, Delavari A, Ansari R, Roshandel G, Merat S, Fitzmaurice C (2019). The global, regional, and national burden of colorectal cancer and its attributable risk factors in 195 countries and territories, 1990–2017: a systematic analysis for the Global Burden of Disease Study 2017. Lancet Gastroenterol Hepatol.

[280] Lorusso G, Rüegg C (2008). The tumor microenvironment and its contribution to tumor evolution toward metastasis. Histochem Cell Biol.

[281] Van Engeland M, Derks S, Smits KM, Meijer GA, Herman JG (2011). Colorectal cancer epigenetics: complex simplicity. J Clin Oncol.

[282] Fearon ER, Vogelstein B (1990). A genetic model for colorectal tumorigenesis. Cell.

[283] Anderson NM, Simon MC (2020). The tumor microenvironment. Curr Biol.

[284] Chen F, Zhuang X, Lin L, Yu P, Wang Y, Shi Y, Hu G, Sun Y (2015). New horizons in tumor microenvironment biology: challenges and opportunities. BMC Med.

[285] Balkwill FR, Capasso M, Hagemann T (2012). The tumor microenvironment at a glance. J Cell Sci.

[286] Zahalka AH, Frenette PS (2020). Nerves in cancer. Nat Rev Cancer.

[287] Albo D, Akay CL, Marshall CL, Wilks JA, Verstovsek G, Liu H, Agarwal N, Berger DH, Ayala GE (2011). Neurogenesis in colorectal cancer is a marker of aggressive tumor behavior and poor outcomes. Cancer.

[288] Liebig C, Ayala G, Wilks J, Verstovsek G, Liu H, Agarwal N, Berger DH, Albo D (2009). Perineural invasion is an independent predictor of outcome in colorectal cancer. J Clin Oncol.

[289] Knijn N, Mogk SC, Teerenstra S, Simmer F, Nagtegaal ID (2016). Perineural invasion is a strong prognostic factor in colorectal cancer: a systematic review. Am J Surg Pathol.

[290] Ayala GE, Dai H, Powell M, Li R, Ding Y, Wheeler TM, Shine D, Kadmon D, Thompson T, Miles BJ, Ittmann MM, Rowley D (2008). Cancer-related axonogenesis and neurogenesis in prostate cancer. Clin Cancer Res.

[291] Kamiya A, Hayama Y, Kato S, Shimomura A, Shimomura T, Irie K, Kaneko R, Yanagawa Y, Kobayashi K, Ochiya T (2019). Genetic manipulation of autonomic nerve fiber innervation and activity and its effect on breast cancer progression. Nat Neurosci.

[292] Zhao C-M, Hayakawa Y, Kodama Y, Muthupalani S, Westphalen CB, Andersen GT, Flatberg A, Johannessen H, Friedman RA, Renz BW, Sandvik AK, Beisvag V, Tomita H, Hara A, Quante M, Li Z, Gershon MD, Kaneko K, Fox JG, Wang TC, Chen D (2014). Denervation suppresses gastric tumorigenesis. Sci Transl Med.

[293] Renz BW, Takahashi R, Tanaka T, Macchini M, Hayakawa Y, Dantes Z, Maurer HC, Chen X, Jiang Z, Westphalen CB, Ilmer M, Valenti G, Mohanta SK, Habenicht AJR, Middelhoff M, Chu T, Nagar K, Tailor Y, Casadei R, Di Marco M, Kleespies A, Friedman RA, Remotti H, Reichert M, Worthley DL, Neumann J, Werner J, Iuga AC, Olive KP, Wang TC (2018). β2 Adrenergic-neurotrophin feedforward loop promotes pancreatic cancer. Cancer Cell.

[294] Hayakawa Y, Sakitani K, Konishi M, Asfaha S, Niikura R, Tomita H, Renz BW, Tailor Y, Macchini M, Middelhoff M, Jiang Z, Tanaka T, Dubeykovskaya ZA, Kim W, Chen X, Urbanska AM, Nagar K, Westphalen CB, Quante M, Lin CS, Gershon MD, Hara A, Zhao CM, Chen D, Worthley DL, Koike K, Wang TC (2017). Nerve growth factor promotes gastric tumorigenesis through aberrant cholinergic signaling. Cancer Cell.

[295] Rademakers G, Vaes N, Schonkeren S, Koch A, Sharkey KA, Melotte V (2017). The role of enteric neurons in the development and progression of colorectal cancer. Biochim Biophys Acta 1868.

[296] Schonkeren SL, Thijssen MS, Vaes N, Boesmans W, Melotte V (2021). The emerging role of nerves and glia in colorectal cancer. Cancers (Basel).

[297] Ratcliffe EM, Fan L, Mohammed TJ, Anderson M, Chalazonitis A, Gershon MD (2011). Enteric neurons synthesize netrins and are essential for the development of the vagal sensory innervation of the fetal gut. Dev Neurobiol.

[298] Mazelin L, Bernet A, Bonod-Bidaud C, Pays L, Arnaud S, Gespach C, Bredesen DE, Scoazec J-Y, Mehlen P (2004). Netrin-1 controls colorectal tumorigenesis by regulating apoptosis. Nature.

[299] Mehlen P, Furne C (2005). Netrin-1: when a neuronal guidance cue turns out to be a regulator of tumorigenesis. Cell Mol Life Sci.

[300] Zhou H, Shi B, Jia Y, Qiu G, Yang W, Li J, Zhao Z, Lv J, Zhang Y, Li Z (2018). Expression and significance of autonomic nerves and α9 nicotinic acetylcholine receptor in colorectal cancer. Mol Med Rep.

[301] Garcia SB, Aranha AL, Garcia FRB, Basile FV, Pinto APM, Oliveira ECd, Zucoloto S (2003). A retrospective study of histopathological findings in 894 cases of megacolon: what is the relationship between megacolon and colonic cancer?. Rev Inst Med Trop Sao Paulo.

[302] Vespúcio MVO, Turatti A, Modiano P, de Oliveira E, Chicote SRM, Pinto A, Garcia SB (2008). Intrinsic denervation of the colon is associated with a decrease of some colonic preneoplastic markers in rats treated with a chemical carcinogen. Braz J Med Biol Res.

[303] Duchalais E, Guilluy C, Nedellec S, Touvron M, Bessard A, Touchefeu Y, Bossard C, Boudin H, Louarn G, Neunlist M (2018). Colorectal cancer cells adhere to and migrate along the neurons of the enteric nervous system. Cell Mol Gastroenterol Hepatol.

[304] Valès S, Bacola G, Biraud M, Touvron M, Bessard A, Geraldo F, Dougherty KA, Lashani S, Bossard C, Flamant M (2019). Tumor cells hijack enteric glia to activate colon cancer stem cells and stimulate tumorigenesis. EBioMedicine.

[305] Seguella L, Rinaldi F, Marianecci C, Capuano R, Pesce M, Annunziata G, Casano F, Bassotti G, Sidoni A, Milone M, Aprea G, de Palma GD, Carafa M, Pesce M, Esposito G, Sarnelli G (2020). Pentamidine niosomes thwart S100B effects in human colon carcinoma biopsies favouring wtp53 rescue. J Cell Mol Med.

[306] Russell JP, Mohammadi E, Ligon CO, Johnson AC, Gershon MD, Rao M, Shen Y, Chan CC, Eidam HS, DeMartino MP, Cheung M, Oliff AI, Kumar S, Greenwood-Van Meerveld B (2019). Exploring the potential of RET kinase inhibition for irritable bowel syndrome: a preclinical investigation in rodent models of colonic hypersensitivity. J Pharmacol Exp Ther.

[307] Chng SH, Pachnis V (2020). Enteric Nervous System: lessons from neurogenesis for reverse engineering and disease modelling and treatment. Curr Opin Pharmacol.

[308] Loffet E, Brossard L, Mahe MM (2020). Pluripotent stem cell derived intestinal organoids with an enteric nervous system. Methods Cell Biol.

[309] Boesmans W, Hao MM, Vanden Berghe P (2018). Optogenetic and chemogenetic techniques for neurogastroenterology. Nat Rev Gastroenterol Hepatol.

